# Mifepristone Promotes Adiponectin Production and Improves Insulin Sensitivity in a Mouse Model of Diet-Induced-Obesity

**DOI:** 10.1371/journal.pone.0079724

**Published:** 2013-11-06

**Authors:** Takeshi Hashimoto, Junsuke Igarashi, Arif U. Hasan, Koji Ohmori, Masakazu Kohno, Yukiko Nagai, Tetsuo Yamashita, Hiroaki Kosaka

**Affiliations:** 1 Department of Cardiovascular Physiology, Faculty of Medicine, Kagawa University, Kagawa, Japan; 2 Department of Cardiorenal and Cerebrovascular Medicine, Kagawa University, Kagawa, Japan; 3 Life Science Research Center, Kagawa University, Kagawa, Japan; University of Lübeck, Germany

## Abstract

The steroid receptor antagonist mifepristone is used as an anti-cancer agent, eliciting both cytostatic and cytotoxic effects on malignant cells. However, the metabolic effects of long-term treatment with mifepristone have remained unclear. The effects of mifepristone on insulin sensitivity and adiponectin secretion were evaluated both in *in vivo* and *in vitro*. First, we explored the effects of mifepristone, on metabolic functions in obese mice receiving a high-fat diet. When these mice were fed mifepristone, they exhibited a marked improvement in insulin sensitivity, attenuated hepatic injury, and decreased adipocyte size, compared with mice that received only the high-fat diet. Intriguingly, mifepristone-treated mice showed significantly elevated plasma adiponectin levels. Second, we tested the effects of mifepristone on differentiated 3T3-L1 adipocytes *in vitro*. When differentiated adipocytes were treated with mifepristone for 48 h, adiponectin was upregulated at both mRNA and protein levels. Collectively, these results reveal novel actions of mifepristone on metabolic functions, *in vivo* and *in vitro*, in which the drug exerts antidiabetic effects associated with an upregulation in adiponectin-secretion.

## Introduction

In the last few decades, a significant amount of research has been dedicated to exploring the complex relationship between obesity and type 2 diabetes mellitus (T2DM) [1,2]. Although understanding this relationship is still a matter of intense study, clinical and basic science research efforts have led to the consensus that obesity can contribute to the development of several health complications, including T2DM, insulin resistance, dyslipidemia and hepatic steatosis. When occurring together, these medical disorders are termed as metabolic syndrome and this has been associated with an increased risk of developing cardiovascular disease and diabetes [3]. Indeed, the current perspective on the etiology of T2DM is that environmental factors, such as high calorie intake, in addition to the genetic abnormalities (i.e., leptin or leptin receptor abnormalities) [4], are considered a major risk factor in the development of obesity-induced T2DM.

Insulin resistance is a key predisposing factor in the pathogenesis of T2DM. Adiponectin is a peptide hormone secreted specifically from adipose tissue. Increasing evidence indicates that adiponectin plays a major role in attenuating insulin resistance. For example, plasma levels of adiponectin are suppressed in obese and diabetic mice compared with healthy littermates [5], as well as in obese humans compared with lean subjects [6]. Conversely, adiponectin supplementation decreases body adiposity and ameliorates insulin resistance that results from either genetic or environmental factors [7]. Pharmacological agents that promote secretion of adiponectin may be candidate therapeutic targets against metabolic disorders including T2DM.

In the early 80s, mifepristone was first introduced as a synthetic steroid with high affinity and antagonistic action at the glucocorticoid receptor (GR) [8]. Although approved as a GR antagonist, mifepristone has been clinically used as an agent for inducing abortion, both for its action as a progesterone receptor antagonist in the luteal phase of the cycle and for early abortion [9]. Recently, experimental studies have revealed that mifepristone treatment decreases blood glucose levels in genetically-modified diabetic mice, suggesting a role for the 11β-hydroxysteroid-dehydrogenase-1 signaling pathway in lowering blood glucose levels [10,11]. However, the mechanisms underlying mifepristone-induced improvement of insulin sensitivity remains less well defined.

 Plasma adiponectin levels are also significantly lower in patients with breast cancer compared with age- and sex- matched healthy women, suggesting a possible association of decreased adiponectin levels with the growth and differentiation of breast epithelial cells and breast cancer cells [12]. These finding are compelling in view of the fact that mifepristone is also used as an anti-cancer agent [9], and suggest the existence of additional unidentified pleiotropic functions. In this study, we will provide evidence that mifepristone augments adiponectin secretion and improves multiple aspects of metabolic functions both in mice *in vivo* and in cultured adipocytes *in vitro*.

## Materials and Methods

### Reagents

Mifepristone and T0070907 were obtained from Cayman Chemical (Ellsworth Road, MI). GW9662 was from MERCK Chemical (Tokyo, Japan). Primer oligonucleotides for real-time quantitative RT-PCR assays (qRT-PCR) were synthesized by Operon Technologies (Tokyo, Japan). FBS was purchased from JRH bioscience (Lenexa, KS). Other reagents were obtained from Sigma (St. Louis, MO) unless otherwise noted.

### Animals and Ethics Statement

All studies were approved by the Kagawa University Institutional Animal Care and Use Committee (IACUC). Six-week-old C57BL/6NCr Slc male mice were purchased from SLC (Shizuoka, Japan). These mice were handled in compliance with the guidelines for conducting Animal Experiments at Kagawa University. All mice were housed one mouse per cage with temperature- and light-control (25°C and 12 h light/12 h dark cycle, respectively). Animals were provided with *ad libitum* access to a regular diet (RD) (Oriental MF diet, Oriental Yeast. Co., Tokyo, Japan) and water for a minimum of 1-week acclimation period before beginning the study. Mice were then divided into five groups and were maintained for 22 weeks on either an RD diet or high-fat diets (HFDs). Mice receiving HFDs were subdivided on the basis of orally administered mifepristone (no mifepristone, 0.1 mg/kg, 1.0 mg/kg, and 30 mg/kg). The maximum concentration of mifepristone in this study was chosen on the basis of an earlier report [13]. The HFD consisted of 35% of calories from fat (powdered beef tallow 15%/100 g and high oleic acid safflower oil 20%/100 g; HFD32, CLEA, Tokyo, Japan)

We measured consumption of food, as well as body weight, every 7 days. Twenty-two weeks after treatment, an insulin tolerance test (ITT) was performed. Following a 7-day recovery period, blood samples were collected, all mice were euthanized, and organ weight was measured.

Liver and white adipose tissues (WAT) were isolated, immediately frozen in liquid nitrogen, and were stored at -80°C until RNA extraction. WAT was taken from the perirenal and epididymal regions. Tissue samples were homogenized using a Beads Cell Disrupter (Micro Smash MS-100, Tomys Seiko Co., Tokyo, Japan) and subjected to column-based RNA isolation procedure as below.

### ITT

 An ITT was performed between 1400 h and 1700 h in mice deprived of food for 6 h before the test. After intraperitoneal injection of human insulin (Humulin, Eli Lilly Japan K.K., Kobe, Japan) (0.75 U/kg body weight), blood samples were collected from the tail vein in series, and blood glucose levels were determined with the use of a Glutest sensor (Sanwakagaku-Arkray, Nagoya, Japan).

### Hematoxylin–eosin staining

Each section from liver and perirenal adipose tissue was fixed in 4% (w/v) paraformaldehyde and embedded in paraffin. 4-µm sections were cut and processed for histopathological examination using hematoxylin and eosin stain. Micrographs were taken using a fluorescent microscope BioRevo BZ9000 (Keyence Japan) and analyzed by using the Keyence software, Dynamic Cell Count.

### Blood sample analysis

Non-fasting serum levels of total serum and the high-molecular-weight (HMW) isoform of adiponectin were measured by enzyme immunoassay (ALPCO Diagnostics, Salem, NH). Serum aspartate aminotransferase (AST) levels were measured by using the Wako Transaminase CII Test (Wako Pure Chemical, Tokyo, Japan).

### RNA preparation and amplification by qRT-PCR

Total RNA was isolated from 3T3-L1 cells and reverse transcribed into first-strand cDNA as we previously reported [14]. Resulting templates were subjected to qRT-PCR and was performed according to manufacturer’s instructions (Roche Diagnostics, Basel, Switzerland), using the TaqMan Universal PCR Master Mix and the Universal ProbeLibrary Probe. Fluorescence-labeled TaqMan MGB probes (200 nM) were used for data collection during the log-linear phase of the reaction. Predesigned primers and probe reagents for mouse adiponectin, leptin, peroxisome proliferator-activated receptor-γ (PPARγ), aP2, and 18S were commercially obtained from Roche. Sequences of the primers and TaqMan probe for detection of mouse transcripts were as follows: adiponectin, forward primer (F): 5′-GGAGAGAAAGGAGATGCAGGT-3′, reverse primer (R): 5′-CTTTCCTGCCAGGGGTTC-3′ and Universal ProbeLibrary probe (UPLP) no. 17; leptin, F: 5′-CAGGATCAATGACATTTCACACA-3′, R: 5′-GCTGGTGAGGACCTGTTGAT-3′ and UPLP no. 93; PPARγ, F: 5′-TTATAGCTGTCATTATTCTCAGTGGAG-3′, R: 5′-GACTCTGGGTGATTCAGCTTG-3′ and UPLP no. 62; aP2, F: 5′-TCGACCACAATAAAGAGAAAACG-3′, R: 5′-CTTGTGGAAGTCACGCCTTT-3′ and UPLP no. 77; and 18S rRNA, F: 5′-CTCAACACGGGAAACCTCAC-3′, R: 5′-CGCTCCACCAACTAAGAACG-3′ and UPLP no. 77. PCR was performed by subjecting the mixtures to activation and denaturation steps for 2 min at 50°C and then for 10 min at 95°C, followed by 40 cycles of 15 s at 95°C, and 1 min at 60°C. Relative degrees of adiponectin, leptin, PPARγ, or aP2 mRNA expression were estimated by normalizing them to those of 18S rRNA in each set of RNA samples.

### Cell culture and treatment with mifepristone

Mouse 3T3-L1 fibroblasts were obtained from JCRB Cell Bank (Osaka, Japan) and were cultured and induced to differentiate as we previously reported [14]. A stock solution of mifepristone was prepared in ethanol at a concentration of 10 mM. Cells were treated either with mifepristone (0.1, 1, or 10 µM) or with vehicle (ethanol).

### Immunoblot analyses (culture medium)

The amount of adiponectin secreted into the culture medium by the 3T3-L1 cells was quantified by Western blot analysis. Cells were cultured in serum-free medium for 3 days. Medium was collected and treated with a same volume of 2x Laemmli sample buffer. The sample loading was based on volume normalization without further sample manipulation such as concentration or precipitation [15]. As controls, we utilized loading Prestained Molecular Weight Markers (cat.7720) (Cell Signaling Technology CST, Beverly, MA).

Each sample was separated by gel electrophoresis and transferred to nitrocellulose membranes (Amersham). Mouse adiponectin antibody (MAB3608) (Millipore, Billerica, MA) was used as the primary antibody. Immunolabeling was visualized using a species-specific horseradish peroxidase (HRP)-conjugated IgG secondary antibody and an enhanced chemiluminescence reagent (SuperSignal West Pico Chemiluminescent Substrate, Pierce, Rockford, IL) using a LAS1000-Plus chemiluminescence image analyzer (Fujifilm Co, Tokyo, Japan).

### Immunoblot analyses (total cellular protein)

 Total cellular proteins were extracted from cultured cells using a Cell Lysis Buffer (cat.9803) (Cell Signaling Technology, Beverly, MA). As previously reported [14], protein quantification was made using a Bicinchoninate Protein Assay Kit (Nacalai, Kyoto, Japan) with bovine serum albumin as a standard. Protein samples were transferred to membranes as above, which were then probed with primary antibodies directed against human adipocyte fatty acid binding protein-2 (aP2) (cat.3544) (Cell Signaling Technology), human PPARγ (cat.2430) (Cell Signaling Technology), mouse Lamin A/C (4C11) (cat.4777) (Cell Signaling Technology), or human β-actin (cat.Ab8229) (Abcam, Cambridge, MA). Immunoreactive signals were visualized as described above. As controls, we utilized loading Prestained Molecular Weight Markers (cat.7720) (Cell Signaling Technology, Beverly, MA).

### [^3^H]-2-deoxy-d-glucose uptake assay in differentiated 3T3-L1 adipocytes

Twelve days after induction of differentiation, mifepristone was added for an additional 3 days. Glucose uptake assay was essentially performed as previously described [16]. 

### Transient transfection with siRNA

The siRNA oligonucleotide sequence targeted to mouse PPARγ was 5′-CUACGACAUGAAUUCCUUAAU-3′ [17]. As a control siRNA, we used MISSION® siRNA Universal Negative Control (Sigma Proligo). 3T3-L1 differentiated adipocytes (12 days after differentiated) were transiently transfected with siRNA duplexes using electroporation (2 nmol, 4-mm cuvette, 950 uF, 250 V) ([18]). Forty-eight hours after transfection, cells were subjected to subcellular fractionation using a commercially available kit [NE-PER® Nuclear and Cytoplasmic Extraction Reagents (Pierce, Cat78833)]. Abundance of PPARγ protein in nuclear and cytosolic fractions were then determined with western blotting as above.

### Statistical analysis

**Figure 1:**
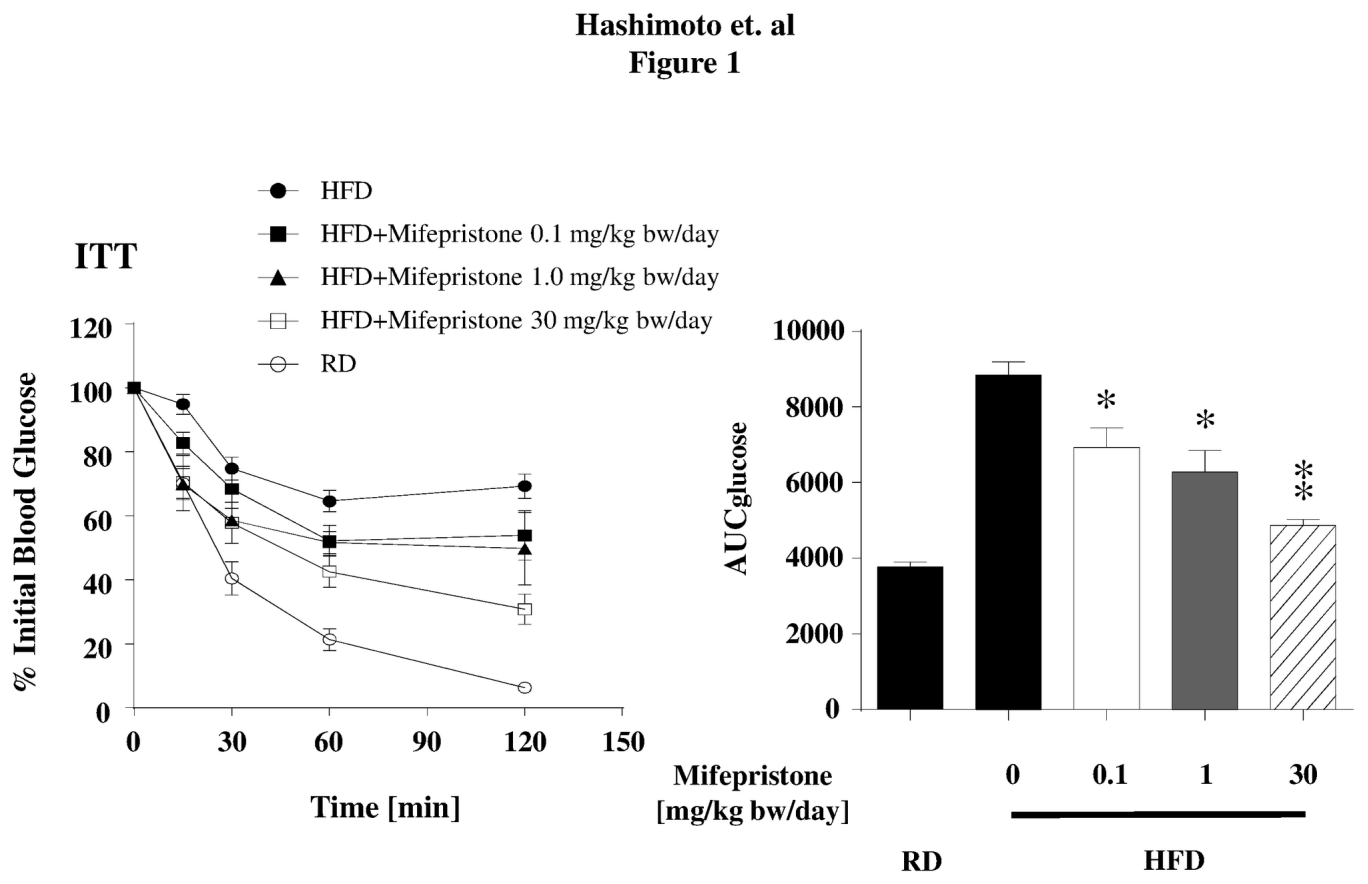
Metabolic effects of mifepristone in HFD induced obese mice. At 28 weeks of age, animals were subjected to an insulin tolerance test (ITT). Left half shows blood glucose values at the time point following insulin administration. Right half summarizes average values of area under the curve (AUC) values obtained from mice with each mifepristone-treatment condition. * *p* < 0.05, ** *p* < 0.01 *versus* HFD fed mice that did not receive mifepristone.

**Figure 2:**
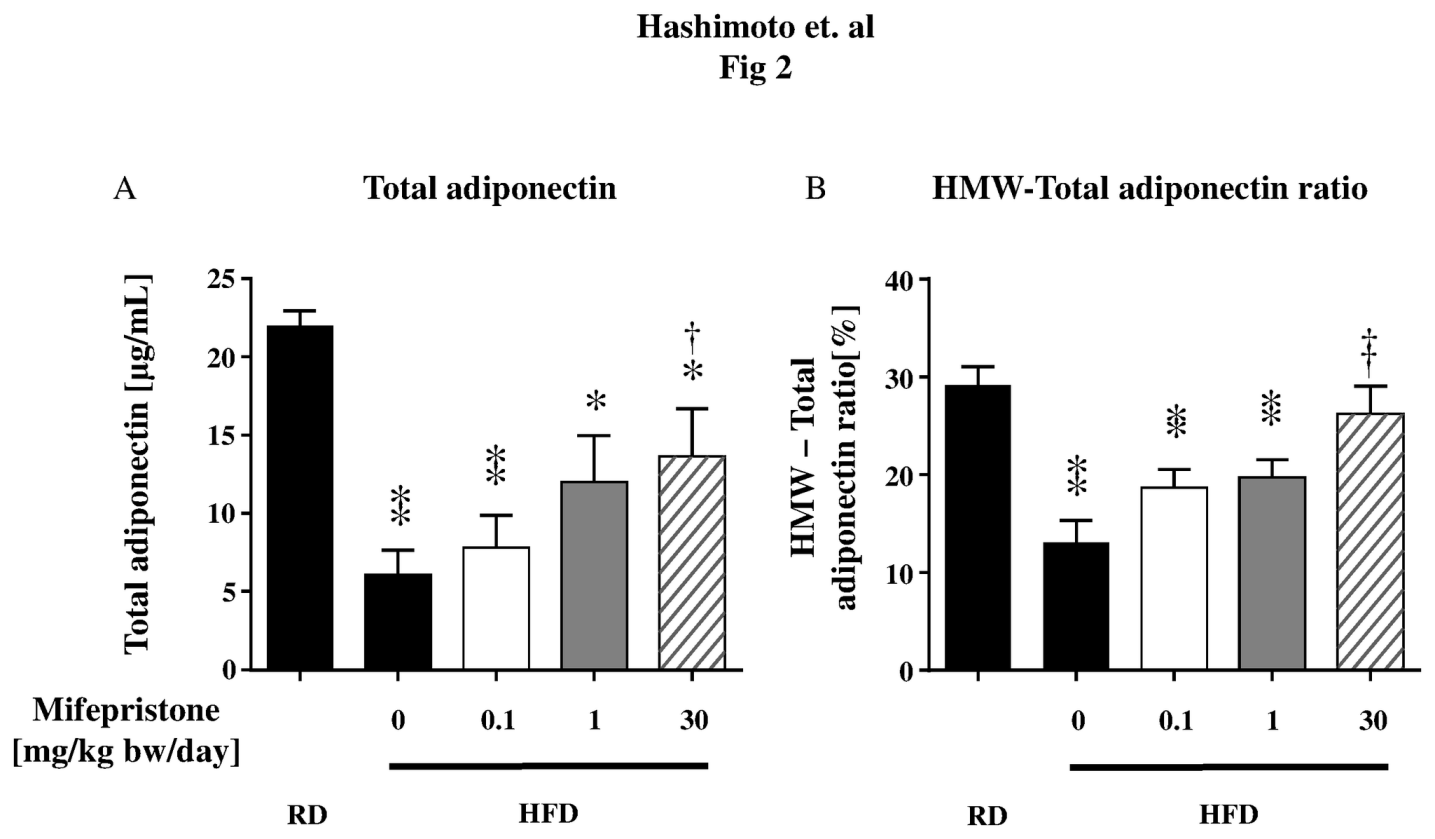
Comparison of serum high-molecular weight (HMW) adiponectin to total adiponectin levels in RD fed mice and HFD-induced obese mice. C57BL/6NCr Slc mice were fed HFD (*n* = 8 in each group). Panel A and B show the changes in total serum and HMW adiponectin levels in mice with HFD-induced obesity. Circulating total and HMW adiponectin levels were determined using an ELISA. Serum samples were collected from age- and sex- matched mice fed with a regular diet (RD). ** *p* < 0.01, * *p* < 0.05 *versus* RD fed mice, † *p* < 0.05, †† *p* < 0.01 *versus* HFD fed mice that did not receive mifepristone.

**Figure 3:**
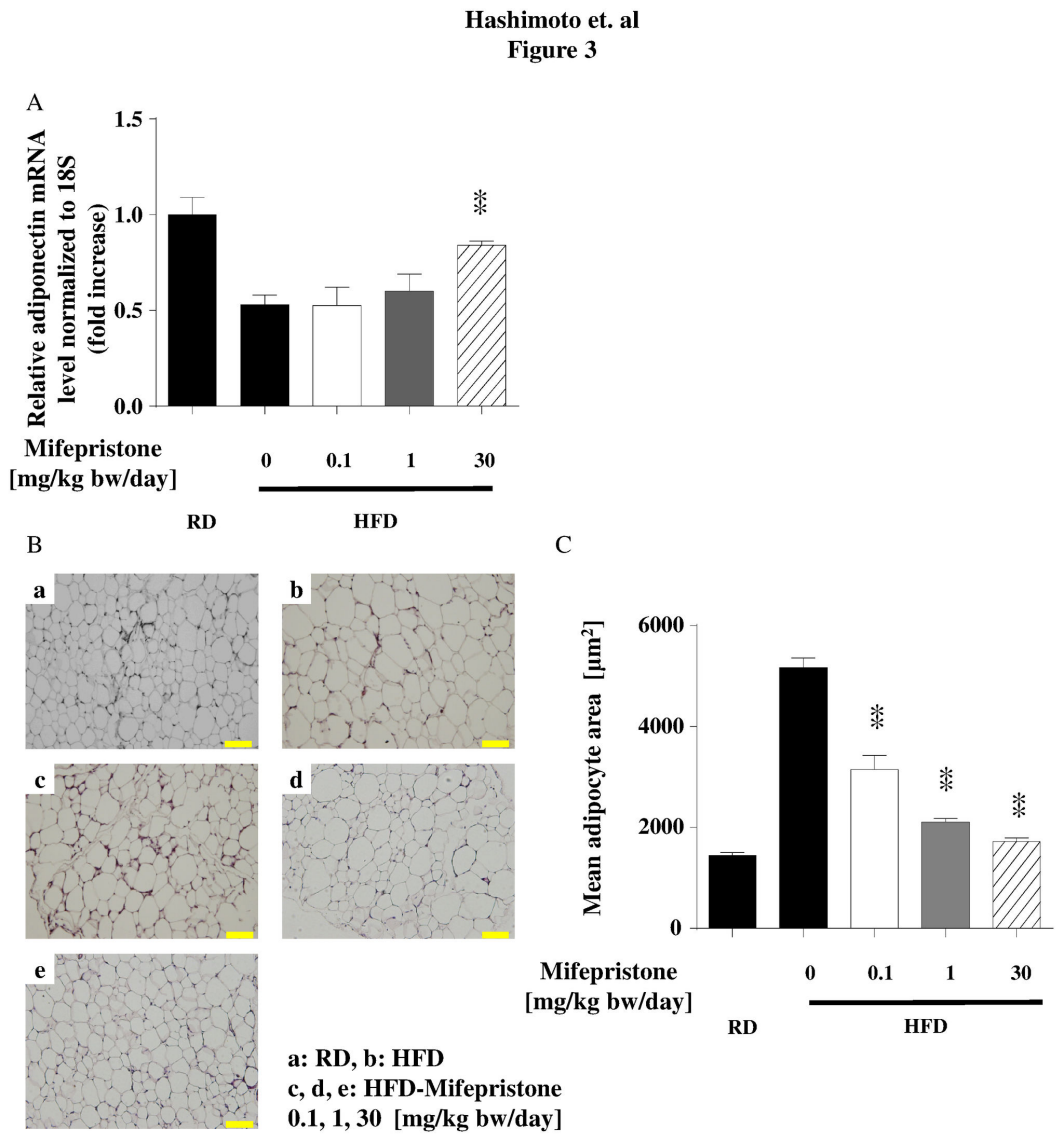
Effects of mifepristone on perirenal adipose tissues in HFD mice. Mice were fed a HFD and treated with mifepristone as described above. Perirenal adipose tissues were collected when the animals were sacrificed. Panel A shows the results of qRT-PCR assays in perirenal adipose tissue taken from mice with HFD-induced obesity treated with or without mifepristone. Total RNA was isolated and subjected to qRT-PCR using primers and probes directed to adiponectin. The expression level of each transcript was normalized to 18S. The results shown represent the pooled data. The fold increase in expression levels of the genes of interest are indicated for each mifepristone concentration, relative to the signals obtained in the HFD alone group. Each data point represents the mean ± S.E.M. derived from eight independent experiments. ** *p* < 0.01 *versus* HFD fed mice that did not receive mifepristone. Panel B shows the results of the histological analysis in perirenal adipose tissues stained with hematoxylin and eosin. Each subpanel demonstrates a representative image obtained from eight animals in each treatment condition. Panel C shows the results of adipose tissue depot quantification in these tissue samples. The adipocyte cross sectional area in each tissue depot was measured as described in the methods; mean values obtained from each mifepristone treatment group are summarized in the graph. ** *p* < 0.01 *versus* HFD fed mice that did not receive mifepristone.

**Figure 4:**
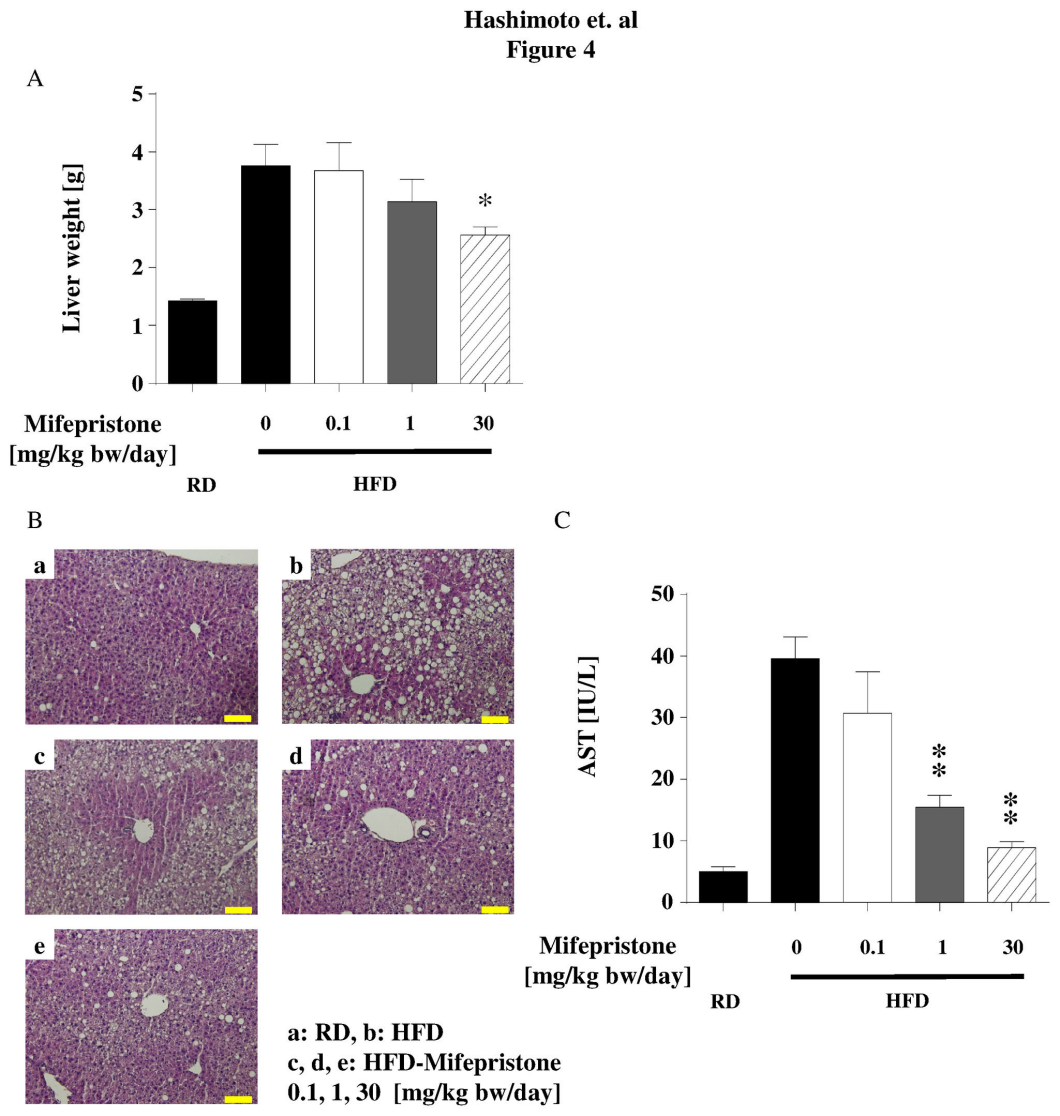
Protective effects of mifepristone on hepatic injury in HFD mice. At 29 weeks of age, mice were euthanized and liver weights were measured (Panel A). Panel B shows liver tissues from mice fed with a HFD and treated with mifepristone. Livers obtained from HFD mice that received various concentrations of mifepristone (or vehicle) as described in Figure 1 were stained with hematoxylin and eosin. Each subpanel demonstrates a representative image obtained from eight animals in each treatment condition. Panel C shows their serum AST levels at the time of sacrifice. * *p* < 0.05, ** *p* < 0.01 *versus* HFD fed mice that did not receive mifepristone.

**Figure 5:**
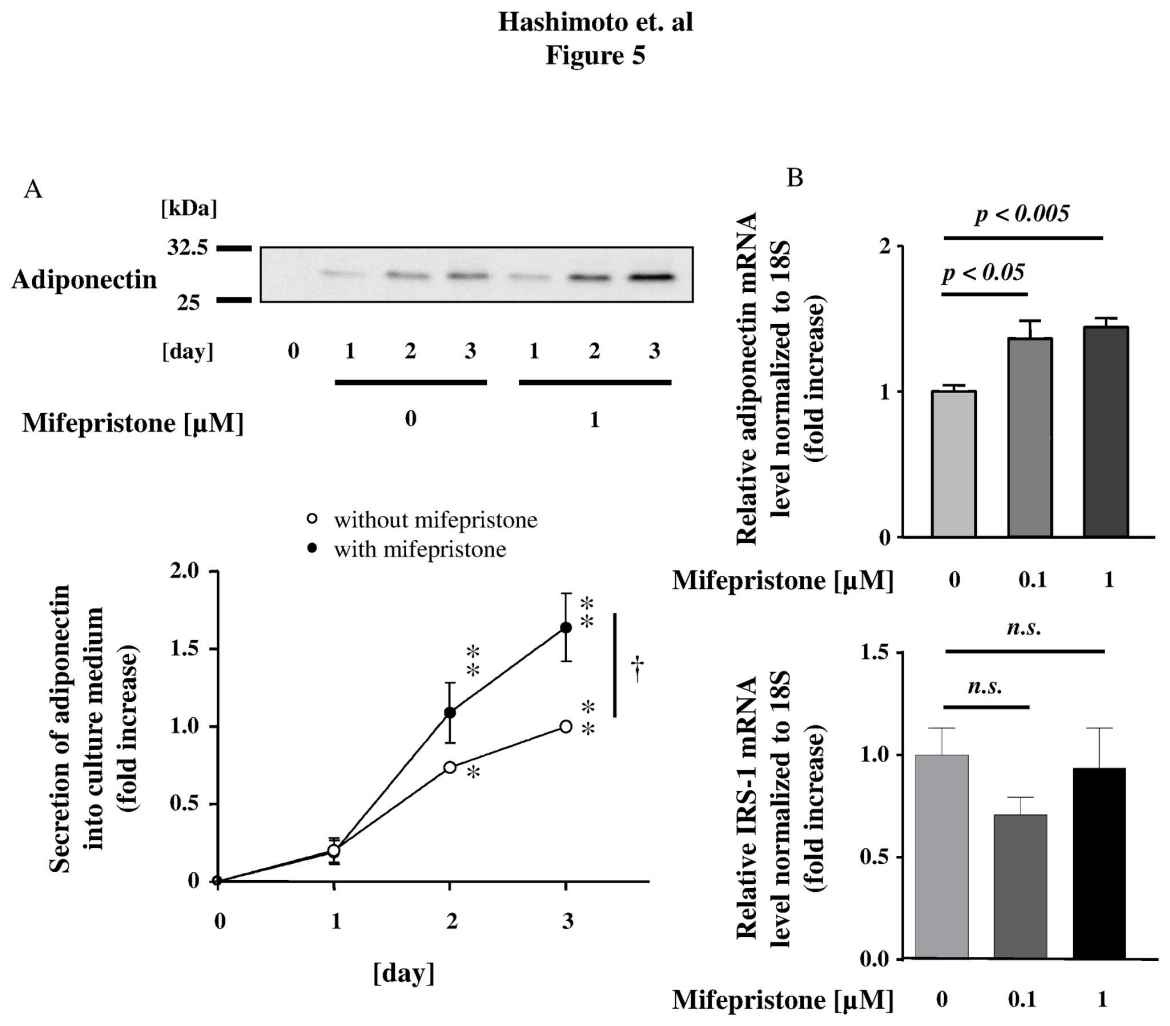
Effects of mifepristone on adiponectin secretion and expression levels in differentiated adipocytes. Shown in the upper half of panel A are the results of the Western blot analysis of the culture medium from differentiated adipocytes stimulated with mifepristone. 3T3-L1 adipocytes were serum starved and treated with 1-µM mifepristone for the times indicated. Equal volumes of culture medium were collected and subjected to immunoblot analysis using an antibody specific to adiponectin. Lower half panel A shows the results of densitometric analysis, plotting the fold increase in the degree of adiponectin secretion levels at the time points after mifepristone addition as indicated. Bands corresponding to adiponectin were quantified by densitometry and were normalized to the signals obtained in the absence of mifepristone (open circle, time = 3 day). Panel B shows the results of qRT-PCR assay. Cells were treated with increasing concentrations of mifepristone for 3 days. RNA was then isolated and qRT-PCR was performed. The expression levels of adiponectin and IRS-1 mRNA were normalized to 18S. Shown are the results derived from pooled data, plotting the fold increase in the degree of adiponectin mRNA expression, relative to the values obtained in the absence of mifepristone. Shown are the mean ± S.E.M. of pooled data, derived from five independent in each group. *n.s.* no significant (*p* > 0.05); * *p* < 0.05, ** *p* < 0.01 *versus* day 1 in each group; † *p* < 0.05 *versus* mifepristone treated cells.

**Figure 6:**
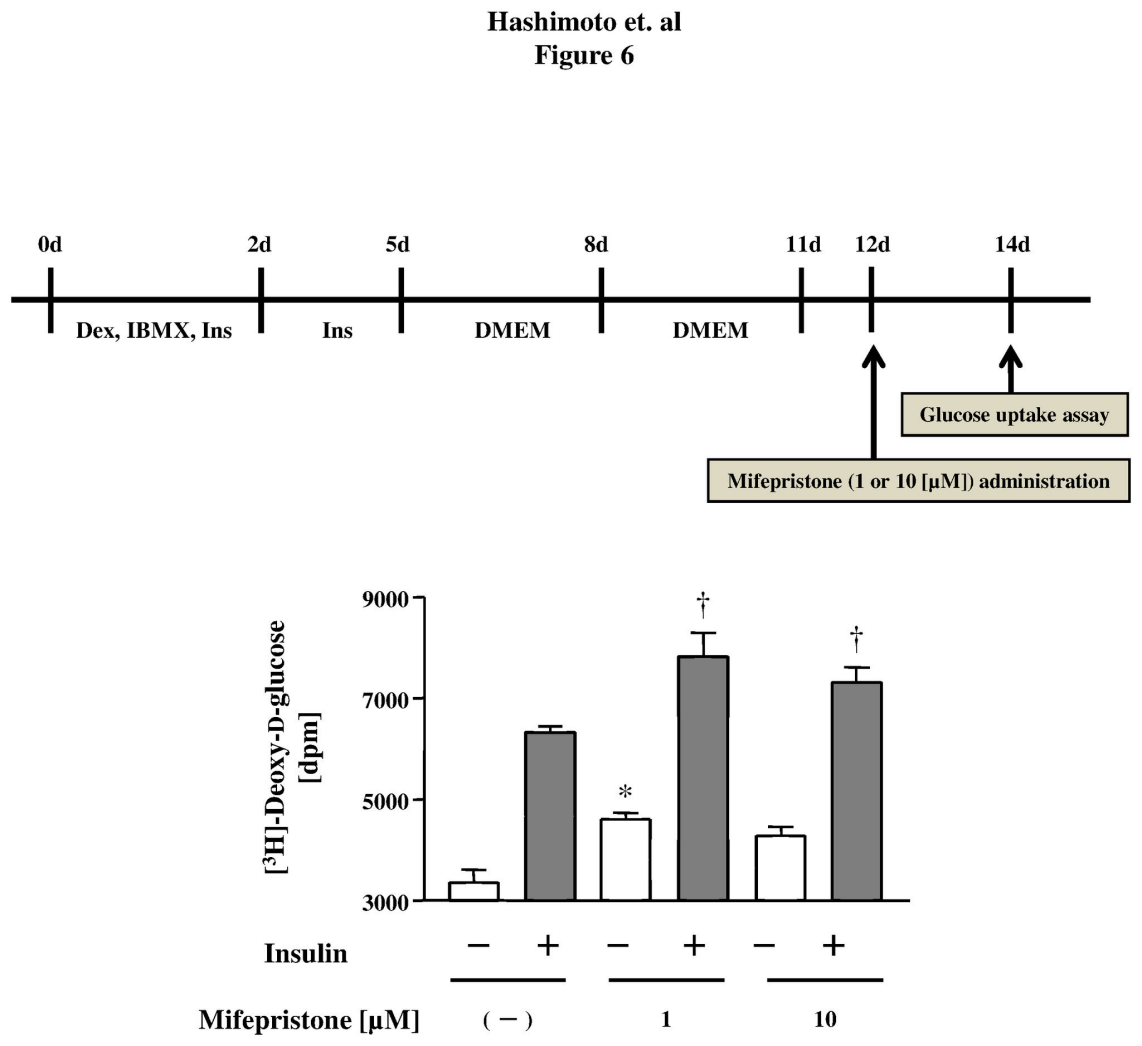
Effects of mifepristone on glucose uptake by mature 3T3-L1 adipocytes. Schedules of the [^3^H]-2-deoxy-d-glucose uptake assay are shown at the top of figure. Cells were treated with mifepristone at the concentrations indicated or with vehicle for 2 days. They were then labeled with [^3^H]-2-deoxy-d-glucose, followed by stimulation with insulin (or vehicle). After cell lysis, they were subjected to liquid scintillation counting. Shown are the mean ± S.E.M. of pooled data, derived from three independent in each group. * *p* < 0.05 *versus* mifepristone (−). † *p* < 0.05 *versus* insulin (−).

**Figure 7:**
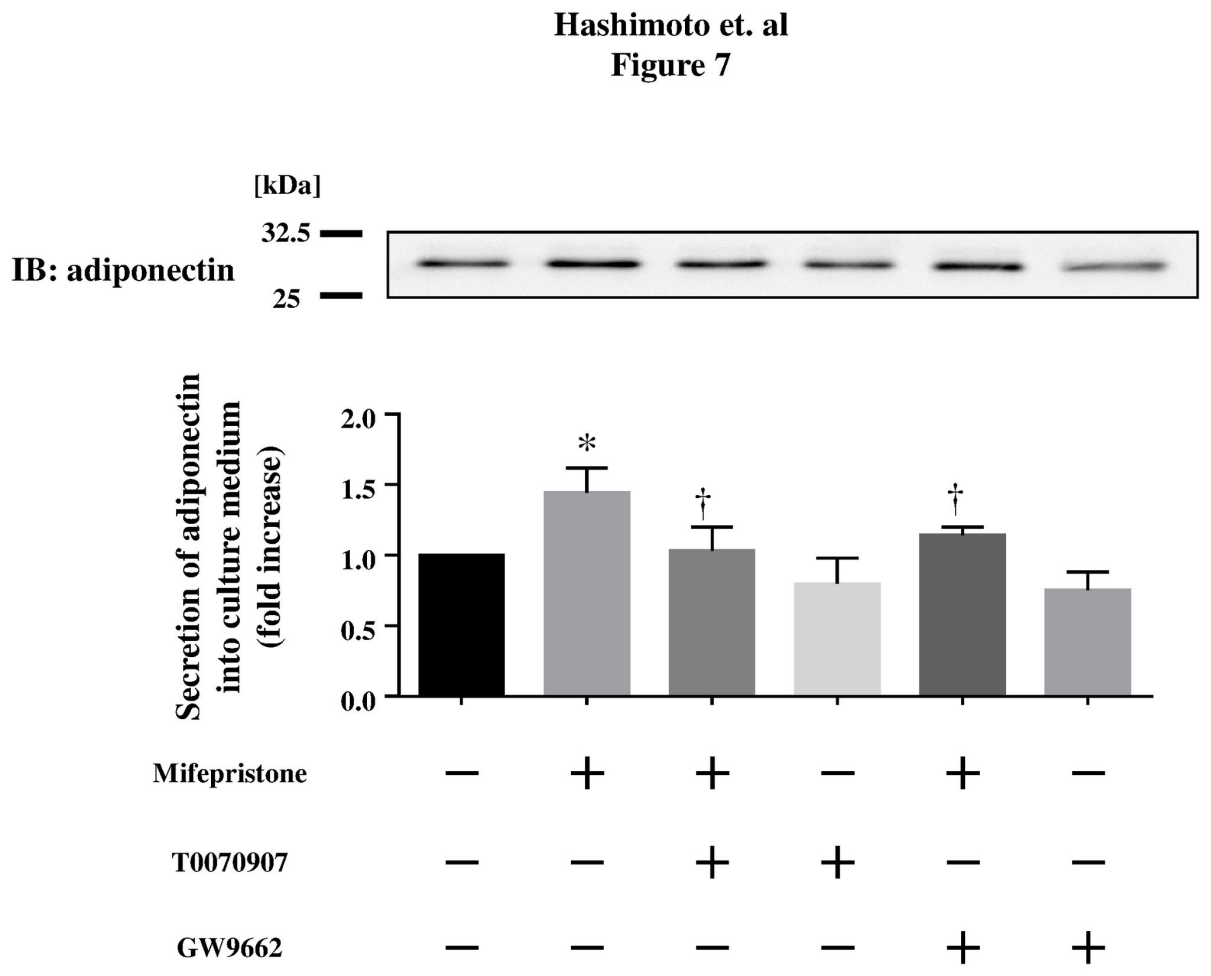
Effects of mifepristone on adiponectin secretion levels in differentiated 3T3-L1 adipocytes. Shown are the results of immunoblot analyses in differentiated 3T3-L1 adipocytes. Cells were treated with mifepristone or vehicle at the indicated concentrations for 3 days. 3T3-L1 adipocytes were serum starved and treated with 1-µM mifepristone and/or 10-µM PPARγ inhibitors (T0070907 or GW9662). Equal volumes of culture medium were collected and analyzed by Western blot analysis using an antibody specific to adiponectin. Lower half panel shows the results of densitometric analysis, plotting the fold increase in the degree of adiponectin secretion levels after mifepristone addition. Bands corresponding to adiponectin were quantified by densitometry and were normalized to the signals obtained in the absence of mifepristone. Pooled data are summarized in the graphs in each assay set. * *p* < 0.05 *versus* for mifepristone compared with vehicle; † *p* < 0.05 *versus* for mifepristone treated cells.

**Figure 8:**
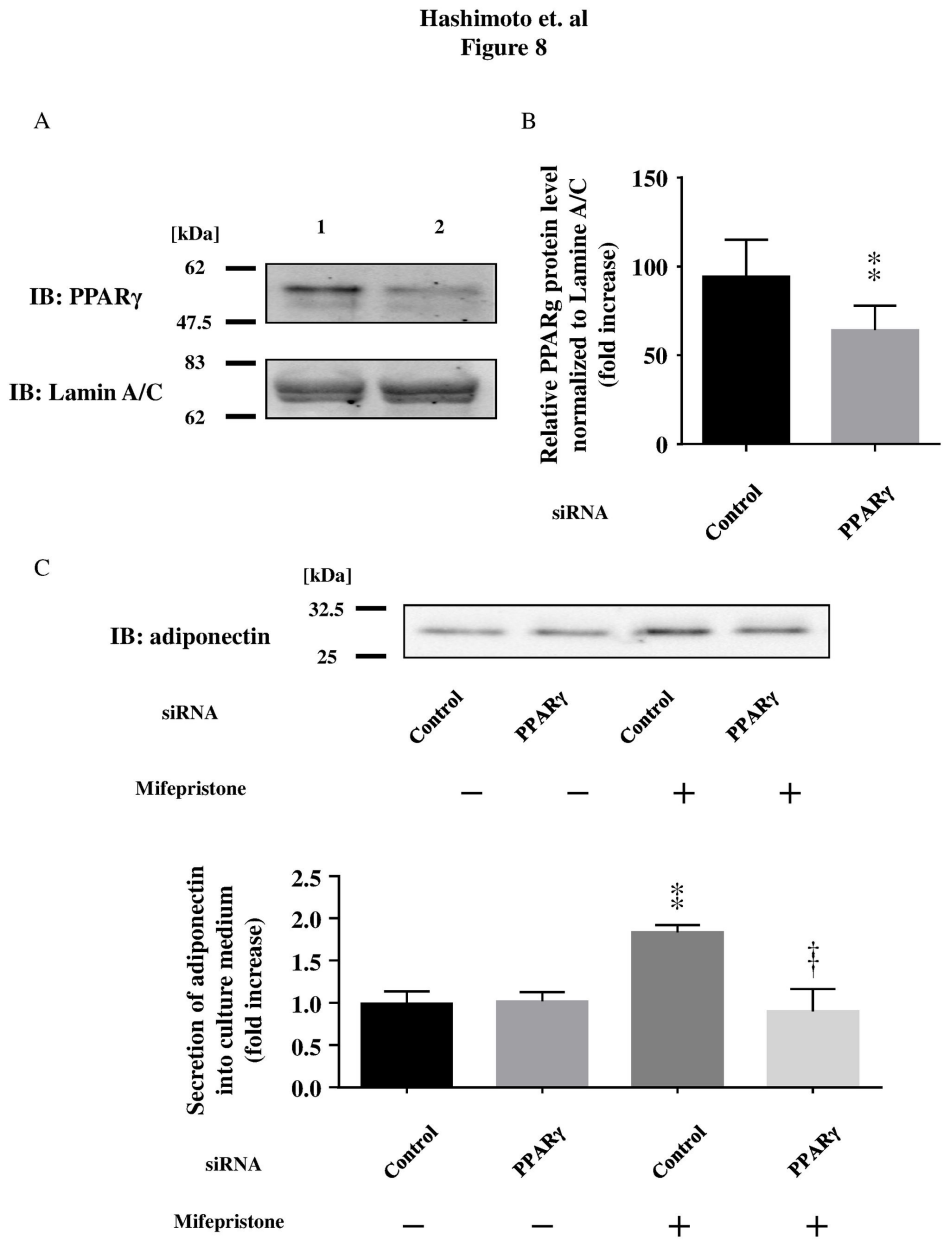
Effects of siRNA targeted to PPARγ in differentiated 3T3-L1 cells. Amounts of PPARγ and Lamine A/C protein in differentiated 3T3-L1 cells after transfection of control siRNA (lane 1) or PPARγ siRNA (lane 2). Lamin A/C is a positive nuclear marker. Data are representative of five experiments (Panel A). Quantification of the PPARγ protein is shown in Panel B using a scanning densitometer. Panel C shows mifepristone-stimulated adiponectin release in differentiated 3T3-L1 adipocytes transfected with either control siRNA or PPARγ siRNA. Pooled data are summarized in the graphs in each assay set. ** *p* < 0.01 *versus* for mifepristone compared with vehicle; †† *p* < 0.01 *versus* for mifepristone treated cells.

Results are expressed as mean ± standard error of mean. The statistical significance of the differences between mean values was determined with an ANOVA followed by Scheffe’s *F* test for multiple groups (Figures 1, 2A, 2B, 3A, 3C, 4A, 4C, 5B, 6, 7, and 8C). ANOVA repeated-measures test was used for the adiponectin secretion analysis (Figure 5A). In Figure 8B, unpaired Student’s *t*-test was performed to analyze the statistical significance of the difference between the two groups. The threshold of significance was set at a *p* value of < 0.05.

## Results

### Effects of mifepristone on HFD-induced insulin resistance *in vivo*

 In order to investigate whether mifepristone influences peripheral insulin-sensitivity and adiponectin secretion, we first explored its effects in HFD-induced obese mice. To this end, we measured the body weight of 7-week-old C57BL/6NCr Slc mice just before the start of the feeding program and randomly assigned eight mice each into to one of the following five groups: RD, HFD alone, or HFD supplemented with 0.1, 1 or 30 mg/kg body weight/day of crude mifepristone. The increase in body weight observed in the HFD groups receiving mifepristone was comparable with that of the HFD alone group, indicating that drug treatment did not affect weight gain (Table 1). Consistent with this, energy intake remained unchanged in mice fed the HFD alone or the HFD treated with concentrations of crude mifepristone (Table 1). However, mifepristone treatment had an effect on fasting blood glucose levels in HFD-induced obese mice. Although fasting blood glucose levels were significantly higher in the HFD-induced obese mice than in the RD-fed mice (Table 1 and Table S1), the HFD mifepristone-treated groups exhibited significantly decreased glucose levels compared with the HFD-alone group (Table 1). Indeed, the fasting blood glucose level in the three HFD groups receiving concentrations of crude mifepristone was not significantly different from the levels observed in the RD mice (Table S1). An ITT revealed that the reduction in blood glucose levels after intraperitoneal administration of insulin was enhanced in all three mifepristone treated groups relative to the HFD alone group (Figure 1, left panel), and this was further supported by a significantly decreased glucose AUC (0-120) (Figure 1, right panel).

**Table 1:** Parameters of high-fat diet-induced obese mice.

HFD + Mifepristone (mg/kg BW/day)	0	0.1	1	30
Total body weight [g]	45.2 ± 1.1	43.8 ± 1.4	44.6 ± 1.1	44.0 ± 1.4
Food consumption [g/day]	3.04 ± 0.04	2.96 ± 0.06	3.04 ± 0.05	3.04 ± 0.04
Fasting blood glucose [mg/dL]	234.3 ± 9.4	190.2 ± 8.7 *	177.7 ± 13.9 **	179.8 ± 14.6 **

Seven-week-old C57BL/6NCr Slc mice received a high-fat diet (HFD) and were orally treated with either mifepristone (0.1, 1 or 30 mg/kg bw/day) or vehicle (HFD alone) for 22 consecutive weeks (*n* = 8 in each group). At the 28 weeks of age, animals were subjected to a fasting blood glucose test. * *p* < 0.05, ** *p* < 0.01 *versus* HFD fed mice that did not receive mifepristone.

 We hypothesized that mifepristone augments responses to insulin in HFD mice by promoting adiponectin secretion. Moreover, because the HMW isoform of adiponectin exerts the most potent effects on blood glucose levels in mice [19], we compared the HMW and the total serum adiponectin levels measured by ELISA. As expected, the HFD-fed group had significantly decrease levels of circulating adiponectin compared with the age-matched RD group (6.7 and 21.9 µg/mL, respectively; Figure 2A). Significantly, treatment with mifepristone induced a recovery in total serum adiponectin levels in a dose-dependent manner (Figure 2A). We also noted that HFD induced a decrease in the total-HMW adiponectin ratio. This decrease was attenuated by the 30 mg/kg bw/day dose of mifepristone (Figure 2B). Indeed, similar to the changes induced in circulating adiponectin, treatment with mifepristone blunted the HFD-induced decrease in adiponectin mRNA production in the perirenal WAT (Figure 3A). In addition, mifepristone upregulated mRNA expression of other molecules known to improve insulin sensitivity, such as aP2, FAS, leptin and PPARγ, in a dose-dependent manner without any effect on the housekeeping gene 18S rRNA in the perirenal WAT (Figure S1).

It has been reported that small-sized adipocytes secrete more insulin-sensitizing adipokines than hypertrophic adipocytes [20]. Therefore, we performed histological studies on the perirenal WAT, to evaluate the impact of mifepristone treatment on adipose tissue morphology. As shown in Figure 3B-a–e, fat pads from the 30-mg/kg bw/day mifepristone group contained a relatively uniform population of small adipocytes compared with the mixed distribution of large and small adipocyte sizes in WAT from HFD mice (Figure 3B-b). Next, we calculated the average cross-sectional area of adipocytes in the perirenal WAT of mice across experimental groups. The mean adipocyte size of the mice fed the HFD supplemented with the three concentrations of crude mifepristone was significantly smaller than that of the mice fed only HFD (Figure 3C, *p < 0.01*).

 Obesity leads to liver injury characterized by steatohepatitis and ballooning degeneration [21], which can be attenuated by administering exogenous adiponectin [22]. We therefore assessed the degree of liver injury in HFD mice treated with or without mifepristone. Liver weight was markedly lower in mice treated with mifepristone at a dose of 30 mg/kg bw/day, despite being unchanged at lower doses (Figure 4A and Figure S2). Histological examination indicated that mice fed the HFD alone exhibited marked ballooning degeneration of hepatocytes, characterized by the presence of numerous vacuoles within the hepatocyte cytoplasm (Figure 4B-b). Interestingly, mifepristone decreased the number of cells undergoing ballooning degeneration in a dose-dependent manner (Figure 4B-c–e). A comparable reduction of serum AST levels was also observed in mifepristone treated mice (Figure 4C). Overall, these findings demonstrate that mifepristone enhances adiponectin secretion from mature adipocytes, which is associated with improvement in insulin sensitivity, amelioration of liver injury, and attenuation of adipocyte hypertrophy.

### The effects of mifepristone in differentiated 3T3-L1 adipocytes *in vitro*

 To gain insight into the mechanisms underlying the above-mentioned effects of mifepristone *in vivo*, we explored the effects of mifepristone on adiponectin secretion from differentiated 3T3-L1 adipocytes cultured in serum-free medium. Mifepristone (1-µM) increased adiponectin secretion by 3T3-L1 adipocytes compared with cells treated with medium alone (Figure 5A). Similarly, adiponectin mRNA expression was also increased by 1.3- or 1.4- fold after 3 days of treatment with 0.1-µM or 1-µM mifepristone, respectively (Figure 5B, upper panel). The effect was also time-dependent; 2 days in vitro, or longer, were required to observe a significant increase in adiponectin mRNA levels (Figure S3). The relative LDH release, i.e., the ratio of LDH released over total LDH, was <0.2% of the total for each incubation (Table S2). Subsequently, we assessed 2-deoxy-d-glucose transport activity in 3T3-L1 adipocytes preincubated with either 1- or 10-µM mifepristone, or vehicle (Figure 6). Insulin caused a 1.9-fold increase in glucose uptake in untreated 3T3-L1 adipocytes, and a significantly greater increase in mifepristone-pretreated cells. Figure 6 summarizes how mifepristone promoted both basal and insulin-stimulated 2-deoxy-d-glucose uptake in these cells. Collectively these experiments show that mifepristone promotes adiponectin secretion and glucose uptake activity in differentiated adipocytes cultured *in vitro*. After finding that mifepristone stimulation elevates adiponectin mRNA expression and secretion by differentiated 3T3-L1 adipocytes, we sought to explore the functional association between adiponectin and the PPARγ pathway. We performed pharmacological experiments in which 3T3-L1 adipocytes were treated with inhibitors of PPARγ, such as T0070907 and GW9662 (up to 10 µM from Day 0 to Day 3 in adipocytes) prior to treating them with mifepristone. As expected, the PPARγ inhibitors markedly attenuated mifepristone-induced adiponectin secretion (Figure 7). Neither T0070907 nor GW9662 altered cell numbers, total protein recovery, or LDH release into culture medium of 3T3-L1 adipocytes after mifepristone stimulation (Table S2, Figure S4). Collectively, these results demonstrate that pharmacological inhibition of the PPARγ pathway attenuates mifepristone-induced adiponectin secretion from 3T3-L1 adipocytes.

As a complementary experiment to the pharmacological studies, we performed a genetic knockdown of PPARγ in differentiated adipocytes. We performed a transient transfection of siRNA against different target sequences of mouse PPARγ mRNA by electroporation. We first attempted to determine the effects of siRNA on endogenously expressed PPARγ protein 2 days after siRNA transfection using the nuclear/cytosol translocation kit. As shown in Figures 8A and 8B, transfection of PPARγ siRNA led to a significant 64 ± 5% reduction in PPARγ protein expression but not Lamine A/C expression, confirming the target specificity of the PPARγ siRNA. Transfection with siRNA specific to PPARγ in 3T3-L1 adipocytes cultured in medium alone did not produce a change in adiponectin release at 3 days *in vitro*, in comparison with 3T3-L1 adipocytes grown in medium alone, and transfected with control siRNA. This indicates that this PPARγ siRNA effectively attenuates mifepristone-induced adiponectin release by differentiated 3T3-L1 adipocytes (Figure 8C). Together, these data indicate that transfection with siRNA targeting mouse PPARγ leads to specific knockdown of PPARγ protein expression as well as marked attenuation of adiponectin release induced by mifepristone stimulation. Off target effects are unlikely because similar effects were obtained in cells transfected with two other distinct siRNA oligonucleotides.

## Discussion

 We have provided experimental results demonstrating that mifepristone, a pharmacological agent previously known as a steroid analogue, promotes secretion of adiponectin both in an *in vivo* model of HFD-induced obesity and in an *in vitro* model of 3T3-L1 cells. Importantly, these effects of mifepristone are associated with improvement of several major metabolic parameters, including insulin tolerance, hepatic injury as well as adipose tissue hypertrophy *in vivo* and glucose uptake *in vitro*.

 Our results demonstrate that increased adiponectin secretion from adipocytes is one of the predominant mechanisms underlying the effects mediated by mifepristone *in vivo*. Previous basic studies using genetically modified mice (*ob/ob* and *db/db*) have reported that short term intraperitoneal injection of mifepristone improved blood sugar levels in the early stages of obesity [10,23]. Subsequently, two clinical studies in healthy male subjects demonstrated that mifepristone significantly attenuated not only the side effect of weight gain caused by the antipsychotic medications olanzapine and risperidone [24,25], but also attenuated the increase in fasting plasma insulin and triglycerides levels caused by the risperidone [25]. Because obesity-induced T2DM is usually caused by an energy imbalance over a prolonged period of time, we first examined the effects of mifepristone in a more relevant long-term HFD-induced obesity mouse model. Mifepristone treatment did not influence energy intake, body weight gain, or body fat percentage (Table 1 and Figure S2). However, mifepristone produced significant protection against insulin resistance in a dose dependent manner (Table 1, Figures 1, 3A, 3B, 3C, 4A, 4B, and 4C). Simultaneous increases in gene expression and serum levels of adiponectin, a well-established antidiabetic adipokine [26] suggests that these mifepristone-mediated effects are in part attributable to an enhancement in adiponectin secretion from adipocytes (Table 1, Figures 2A, 2B, 3A, 3B, 3C, and Table S1). Of note, the effects of mifepristone in the ITT are statistically more robust at the 30-mg/kg bw/day treatment group than at the 1 or 0.1 mg/kg (γ). This may be partly explained by the fact that the drug elevated HMW-to-total adiponectin levels at the 30 mg/kg bw/day treatment, only (Figure 2B). It will be more important to rigorously determine the necessary amounts of mifepristone to improve metabolic parameters and/or adiponectin secretion using multiple experimental models. Glucose tolerance tests (GTT) in mifepristone-treated animals may provide further insights as well.

It is now well established that insulin sensitizing drugs such as thiazolidinedione produce their effect through body-wide repartitioning of lipid storage and by altering signaling molecules such as adiponectin [27]. Notably, we have observed a significant reduction in hepatocyte ballooning and hepatic steatosis by mifepristone treatment (Figure 4B), suggesting enhanced lipid mobilization in this organ. Moreover, adiponectin has been shown to ameliorate insulin resistance through insulin receptor substrates (IRSs) in the liver [28]. Although we did not directly examine the effects of mifepristone on IRS in the current study, either at the level of protein expression or tyrosine phosphorylation, enhanced secretion of serum adiponectin from adipose tissues suggests that mifepristone exerts its effects through their remote activity [27]. However, further studies are required to understand the role of mifepristone in the liver exposed to a HFD.

The mechanisms underlying mifepristone-mediated improvement of insulin sensitivity remain to be fully elucidated. We have observed an enhanced expression of PPARγ, aP2, FAS, and leptin, which are genes known to promote lipid storage and lipogenesis [29] in adipose tissues of mifepristone-treated mice. Similarly, basal adiponectin secretion from differentiated 3T3-L1 adipocytes was significantly increased by the addition of mifepristone to the culture medium, which is in agreement with an enhancement of adiponectin mRNA production (Figure 5B, upper panel). Note that the expression levels of IRS-1 mRNA remain unchanged in these cells following mifepristone stimulation (Figure 5B, lower panel). Although IRS-1 signaling is regulated at multiple levels including tyrosine phosphorylation [30], we speculate that mifepristone modulates metabolic functions without altering the expression levels of IRS-1. Indeed, identification of the molecular mechanisms underlying the effects of mifepristone on metabolic functions remains to be resolved. We tested several other pharmacological agents chemically or pharmacologically related to mifepristone: GR antagonists, pregnenolone and DHEA; synthetic progestin causing abortion, levonorgestrel; MR antagonist, spironolactone [8,31–34]. None of these drugs swere capable of promoting adiponectin secretion (*Hashimoto et al. unpublished observation*). We therefore propose that the ability to enhance adiponectin secretion is a function that is specific to mifepristone, rather than representing a class effect. Our data indicate that pharmacological (T0070907 and GW9662) as well as genetic (siRNA) inhibition of PPARγ is able to attenuate mifepristone-induced adiponectin secretion by differentiated 3T3-L1 adipocytes *in vitro*, suggesting that mifepristone exerts its effects through the PPARγ pathway.

Moreover, a recent study has reported that mifepristone can bind the PPARγ ligand-binding pocket [35]. Interestingly, we have observed increased PPARγ expression along with its downstream target aP2 gene in both mice and cell culture studies (Figure S1 and *Hashimoto et al. unpublished observation*). Although there is evidence that adiponectin can modulate cellular functions in both PPARγ-dependent and -independent fashions [36], we speculated that PPARγ might be involved in upregulating adiponectin, a molecule that when suppressed is linked to insulin resistance [37,38]. It is of note that an earlier study reported that the PPARγ agonist, pioglitazone improves HFD-induced insulin resistance and steatohepatitis in rodents, whereas body weight is still increased [39]..Consistent with this, our data indicate that mifepristone improves insulin sensitivity without affecting total body weight. Thus, mifepristone and classical PPARγ agonists may modulate insulin sensitivity and whole body weight in distinct manners. Unraveling the mechanisms underlying the effects of mifepristone on these and other adipocyte signaling pathways would clearly benefit our understanding of the pathophysiology of metabolic disorders and provide novel therapeutic pharmacological targets.

Furthermore, mifepristone augmented 2-deoxy-d-glucose uptake both in the basal and insulin-stimulated state of 3T3-L1 adipocytes (Figure 6). A previous report has shown that the potency of these insulin-sensitizing effects is comparable with what is observed in adiponectin-treated cultured adipocytes [40]. It is important to mention that treatment with mifepristone does not lead to any observable variations in LDH release, cellular protein level or cell number (Table S2, Figures S5, and S6). Collectively, these results support our hypothesis that both mRNA expression and secretion of adiponectin are induced by mifepristone in adipocytes.

Our *in vivo* and *in vitro* results show that treatment with mifepristone leads to an increase in the proportion of small adipocytes, which likely reflects enhanced adipocyte proliferation and/or suppressed adipocyte hypertrophy. A similar effect has been reported for thiazolidinedione, a PPARγ agonist, and its impact on adipocyte function is considered to be an additional mechanism by which this agent improves insulin sensitivity [41]. As a previous report has shown that thiazolidinedione enhances preadipocyte proliferation [42], the number of adipocytes in perirenal adipose tissue may be elevated by mifepristone. However, in our *in vitro* studies using differentiated 3T3-L1 adipocytes, treatment with mifepristone does not lead to any observable variations in the cell number detected by DAPI stained nuclei (Figure S5). Moreover, mifepristone did not enhance or suppress preadipocyte proliferation of 3T3-L1 cells during adipocyte differentiation (*Hashimoto et al. unpublished observation*). We speculate that these seemingly contradictory findings stem from many differences in assay conditions between our *in vivo* and *in vitro* experiments; for example, the *in vivo* assays were performed over a much longer period than the *in vitro* assays (30 weeks versus 3 days). Thus, our data highlight the differential activity of mifepristone, which exerts anti-proliferative actions in cultured breast cancer cells [43,44], and effects on the metabolic actions in adipose tissues and adipocytes. It is therefore plausible that the effect of mifepristone on cell proliferation is highly cell-specific, remarkably reminiscent of the effect of thiazolidinedione [45].

 It is of note that mifepristone is able to promote secretion of adiponectin from fully differentiated adipocytes (Figure 5A and Figure S6). In these experiments we used serum-free medium because FBS contains endogenous circulating adiponectin [46], which makes it difficult to discriminate 3T3-L1 cell-derived molecules (Figure S6). Note that serum-free medium and transfection of siRNAs did not alter LDH release, cellular protein levels, or the morphology of these cells (during 3-day) (Figures S5, S7, and S8 as well as Table S2). Mifepristone, in contrast, modestly increased the secretion ability of adiponectin into the serum-free medium (Figure 5A and Figure S6). Although, we focused on PPARγ in our experiments with siRNA, more detailed quantitative characterizations of both types of PPARγ, in terms of adiponectin-producing capacity as well as functional regulation of adipose tissues and differentiated adipocytes, remain to be elucidated. However, in both cases, mifepristone is capable of augmenting adiponectin secretion by cultured 3T3-L1 cells, as revealed by immunoblot analysis. When combined with our adiponectin data *in vivo* (Figures 2A and 3A) and qRT-PCR data *in vitro* (Figure 5B), these data point to mifepristone as a novel agent that promotes adiponectin production from adipose tissues.

In conclusion, we have demonstrated that mifepristone induces expression of adiponectin in adipose tissue from mice with HFD-induced obesity as well as in differentiated mouse 3T3-L1 adipocytes, and that these are associated with improvements in several key metabolic parameters. We propose that a deeper understanding of the relationship between mifepristone and adiponectin signaling in adipose tissue may lead to the identification of novel therapeutic targets in obesity-induced T2DM along with other potentially related pathophysiological conditions, including breast cancer and metabolic diseases.

## Supporting Information

Figure S1 **Effects of mifepristone on perirenal adipose tissues in HFD mice.** Mice had been fed with HFD and mifepristone as described above. Perirenal adipose tissues was collected when the animals were sacrificed. Panel shows the results of qRT-PCR assays in perirenal adipose tissue of HFD-induced obesity mice treated with or without mifepristone. Total RNA was isolated and subjected to qRT-PCR using primers and probes directed to aP2, FAS, leptin, and PPARγ. Expression level of each transcript was normalized to that of 18S. Shown are the results derived from pooled data, plotting the fold increase of the expression levels of indicated gene at the mifepristone-concentration indicated, relative to the signals obtained in the absence of mifepristone (HFD alone). Each data represents the mean ± S.E.M. derived from 8 independent experiments. * *p* < 0.05, ** *p* < 0.01 *versus* HFD fed mice that did not receive mifepristone.
(PPT)

Figure S2 **Effects of mifepristone on percent of total body weight (Liver and Perirenal adipose tissue) in HFD mice.** Tissue weights (liver and perirenal adipose tissues) in HFD induced obese mice are expressed as percentage of body weight. Each data represents the mean ± S.E.M. derived from 8 independent experiments. * *p* < 0.05 *versus* HFD fed mice that did not receive mifepristone.
(PPT)

Figure S3 **Effects of mifepristone on matured adipocytes.** Shown are the results of qRT-PCR and immunoblot analysis. Cells were treated with 0.1 and 1 µM of mifepristone for the times indicated. Upper panel shows that the expression level of adiponectin mRNA was normalized to that of 18S, respectively. Shown are the results derived from pooled data, plotting the fold increase of the degree of expression level of adiponectin mRNA, relative to the values obtained in the absence of mifepristone (day 0). Each data represents the mean ± S.E.M. derived from 4 independent experiments. * *p* < 0.05, ** *p* < 0.01 *versus* the absence of mifepristone. Lower panel shows adiponectin secretion levels from cells kept for 3 days after indicated concentration of mifepristone.
(PPT)

Figure S4 **Effects of mifepristone and PPARγ antagonists on total cellular protein level in matured adipocytes.** Shown are the results of total cellular protein. Cells were treated with 0.1 and 1 µM of mifepristone. Total cellular proteins were extracted using a buffer containing n-octyl b-glucopyranoside. Protein determinations were made with Bicinchoninate Protein Assay Kit (Nacalai, Kyoto, Japan) with bovine serum albumin as a standard. Shown are the results derived from pooled data, relative to the values obtained in the absence of mifepristone (day 3). Each data represents the mean ± S.E.M. derived from 4 independent experiments.
(PPT)

Figure S5 **Effects of mifepristone on cell number in matured adipocytes.** The degrees of cell number were determined using 4′,6-diamidino-2-phenylindole (DAPI) solution, visualized nuclear DNA. Cells were fixed, stained and observed by microscope (BioRevo, KEYENCE). Shown are the results derived from pooled data, relative to the values obtained in the absence of mifepristone (day 3). Each data represents the mean ± S.E.M. derived from 3 independent experiments. Bar, 100 microm.
(PPT)

Figure S6 **Immunoblot analysis of adiponectin secretion levels into culture medium (DMEM) with or without fetal bovine serum (FBS).** Cells were kept for 3 days after mifepristone stimulation, then equal volume of medium containing 2x Laemmli sample buffer was added. * p < 0.05 *versus* vehicle, Each value and vertical bar represents the mean±SE (n = 10).
(PPT)

Figure S7 **Differentiated 3T3-L1 adipocytes had been transiently transfected with control or siRNA targeted to PPARγ 48 hours prior to mifepristone stimulation.** Cells were fixed and observed by microscope (BioRevo, KEYENCE). Bar, 100 microm.
(PPT)

Figure S8 **Effects of mifepristone on total cellular protein level in matured adipocytes transfected with each siRNA.** Shown are the results of total cellular protein. Cells were transfected with siRNA for control or PPARγ treated with/without mifepristone. Total cellular proteins were extracted using a buffer containing n-octyl b-glucopyranoside. Protein determinations were made with Bicinchoninate Protein Assay Kit (Nacalai, Kyoto, Japan) with bovine serum albumin as a standard. Shown are the results derived from pooled data, relative to the values obtained in the absence of mifepristone (day 3). Each data represents the mean ± S.E.M. derived from 4 independent experiments.
(PPT)

Table S1 **Parameters of regular diet mice.** Seven-week-old C57BL/6NCr Slc mice received regular diet (RD) and were orally treated with either mifepristone (0.1, 1 or 30 mg/kg bw/day) or vehicle (RD alone) for twenty-two consecutive weeks (*n* = 8 in each group). At the age of week 28, animals were subjected to fasting blood glucose test. * *p* < 0.05, ** *p* < 0.01 *versus* RD fed mice that did not receive mifepristone.
(PPT)

Table S2 **LDH activity assay.** LDH activity of culture medium was measured using a CytoTox-ONE Homogenous Membrane Integrity Assay kit (Promega), using DMEM and cell lysates extracted with a lysis buffer containing Triton-X (0.1% v/v) as negative and positive controls, respectively.
(PPT)

## References

[1] Lumeng CN, Saltiel AR (2011) Inflammatory links between obesity and metabolic disease. J Clin Invest 121(6): 2111-2117. doi:10.1172/JCI57132. PubMed: 21633179. 21633179 PMC3104776

[2] Seino S, Shibasaki T, Minami K (2011) Dynamics of insulin secretion and the clinical implications for obesity and diabetes. J Clin Invest 121(6): 2118-2125. doi:10.1172/JCI45680. PubMed: 21633180. 21633180 PMC3104758

[3] Sharma AM (2006) The obese patient with diabetes mellitus: from research targets to treatment options. Am J Med 119 Suppl 1)(5: S17-S23. doi:10.1016/j.amjmed.2005.10.044. PubMed: 16563943. 16563943

[4] O'Rahilly S, Barroso I, Wareham NJ (2005) Genetic factors in type 2 diabetes: the end of the beginning? Science 307(5708): 370-373. doi:10.1126/science.1104346. PubMed: 15662000. 15662000

[5] Lagathu C, Yvan-Charvet L, Bastard JP, Maachi M, Quignard-Boulangé A et al. (2006) Long-term treatment with interleukin-1beta induces insulin resistance in murine and human adipocytes. Diabetologia 49(9): 2162-2173. doi:10.1007/s00125-006-0335-z. PubMed: 16865359. 16865359

[6] Kern PA, Di Gregorio GB, Lu T, Rassouli N, Ranganathan G (2003) Adiponectin expression from human adipose tissue: relation to obesity, insulin resistance, and tumor necrosis factor-alpha expression. Diabetes 52(7): 1779-1785. doi:10.2337/diabetes.52.7.1779. PubMed: 12829646. 12829646

[7] Berg AH, Combs TP, Scherer PE (2002) ACRP30/adiponectin: an adipokine regulating glucose and lipid metabolism. Trends Endocrinol Metab 13(2): 84-89. doi:10.1016/S1043-2760(01)00524-0. PubMed: 11854024. 11854024

[8] Schreiber JR, Hsueh AJ, Baulieu EE (1983) Binding of the anti-progestin RU-486 to rat ovary steroid receptors. Contraception 28(1): 77-85. doi:10.1016/S0010-7824(83)80008-0. PubMed: 6627946. 6627946

[9] Cadepond F, Ulmann A, Baulieu EE (1997) RU486 (mifepristone): mechanisms of action and clinical uses. Annu Rev Med 48: 129-156. doi:10.1146/annurev.med.48.1.129. PubMed: 9046951. 9046951

[10] Taylor AI, Frizzell N, McKillop AM, Flatt PR, Gault VA (2009) Effect of RU486 on hepatic and adipocyte gene expression improves diabetes control in obesity-type 2 diabetes. Horm Metab Res 41(12): 899-904. doi:10.1055/s-0029-1234071. PubMed: 19670152. 19670152

[11] Wang Y, Nakagawa Y, Liu L, Wang W, Ren X et al. (2011) Tissue-specific dysregulation of hexose-6-phosphate dehydrogenase and glucose-6-phosphate transporter production in db/db mice as a model of type 2 diabetes. Diabetologia 54(2): 440-450. doi:10.1007/s00125-010-1956-9. PubMed: 21052977. 21052977 PMC3795617

[12] Kang JH, Lee YY, Yu BY, Yang BS, Cho KH et al. (2005) Adiponectin induces growth arrest and apoptosis of MDA-MB-231 breast cancer cell. Arch Pharm Res 28(11): 1263-1269. doi:10.1007/BF02978210. PubMed: 16350853. 16350853

[13] Kusunoki M, Cooney GJ, Hara T, Storlien LH (1995) Amelioration of high-fat feeding-induced insulin resistance in skeletal muscle with the antiglucocorticoid RU486. Diabetes 44(6): 718-720. doi:10.2337/diabetes.44.6.718. PubMed: 7789638. 7789638

[14] Hashimoto T, Igarashi J, Kosaka H (2009) Sphingosine kinase is induced in mouse 3T3-L1 cells and promotes adipogenesis. J Lipid Res 50(4): 602-610. PubMed: 19020339. 19020339 10.1194/jlr.M800206-JLR200 PMC2656653

[15] Maeda N, Takahashi M, Funahashi T, Kihara S, Nishizawa H et al. (2001) PPARgamma ligands increase expression and plasma concentrations of adiponectin, an adipose-derived protein. Diabetes 50(9): 2094-2099. doi:10.2337/diabetes.50.9.2094. PubMed: 11522676. 11522676

[16] Hitomi H, Kiyomoto H, Nishiyama A, Hara T, Moriwaki K et al. (2007) Aldosterone suppresses insulin signaling via the downregulation of insulin receptor substrate-1 in vascular smooth muscle cells. Hypertension 50(4): 750-755. doi:10.1161/HYPERTENSIONAHA.107.093955. PubMed: 17646573. 17646573

[17] Liao W, Nguyen MT, Yoshizaki T, Favelyukis S, Patsouris D et al. (2007) Suppression of PPAR-gamma attenuates insulin-stimulated glucose uptake by affecting both GLUT1 and GLUT4 in 3T3-L1 adipocytes. Am J Physiol Endocrinol Metab 293(1): E219-E227. doi:10.1152/ajpendo.00695.2006. PubMed: 17389706. 17389706

[18] Liao W, Nguyen MT, Imamura T, Singer O, Verma IM et al. (2006) Lentiviral short hairpin ribonucleic acid-mediated knockdown of GLUT4 in 3T3-L1 adipocytes. Endocrinology 147(5): 2245-2252. doi:10.1210/en.2005-1638. PubMed: 16497797. 16497797

[19] Pajvani UB, Hawkins M, Combs TP, Rajala MW, Doebber T et al. (2004) Complex distribution, not absolute amount of adiponectin, correlates with thiazolidinedione-mediated improvement in insulin sensitivity. J Biol Chem 279(13): 12152-12162. PubMed: 14699128. 14699128 10.1074/jbc.M311113200

[20] Kadowaki T, Hara K, Yamauchi T, Terauchi Y, Tobe K et al. (2003) Molecular mechanism of insulin resistance and obesity. Exp Biol Med (Maywood) 228(10): 1111-1117. PubMed: 14610248. 14610248 10.1177/153537020322801003

[21] Lee YM, Choi JS, Kim MH, Jung MH, Lee YS et al. (2006) Effects of dietary genistein on hepatic lipid metabolism and mitochondrial function in mice fed high-fat diets. Nutrition 22(9): 956-964. doi:10.1016/j.nut.2005.12.014. PubMed: 16814985. 16814985

[22] Xu A, Wang Y, Keshaw H, Xu LY, Lam KS et al. (2003) The fat-derived hormone adiponectin alleviates alcoholic and nonalcoholic fatty liver diseases in mice. J Clin Invest 112(1): 91-100. doi:10.1172/JCI17797. PubMed: 12840063. 12840063 PMC162288

[23] Yang B, Trump RP, Shen Y, McNulty JA, Clifton LG et al. (2008) RU486 did not exacerbate cytokine release in mice challenged with LPS nor in db/db mice. BMC Pharmacol 8: 7. doi:10.1186/1471-2210-8-S1-A7. PubMed: 18474108. 18474108 PMC2396158

[24] Gross C, Blasey CM, Roe RL, Allen K, Block TS et al. (2009) Mifepristone treatment of olanzapine-induced weight gain in healthy men. Adv Ther 26(10): 959-969. doi:10.1007/s12325-009-0070-1. PubMed: 19888560. 19888560

[25] Gross C, Blasey CM, Roe RL, Belanoff JK (2010) Mifepristone reduces weight gain and improves metabolic abnormalities associated with risperidone treatment in normal men. Obesity (Silver Spring) 18(12): 2295-2300. doi:10.1038/oby.2010.51. PubMed: 20339369. 20339369

[26] Yamauchi T, Kamon J, Ito Y, Tsuchida A, Yokomizo T et al. (2003) Cloning of adiponectin receptors that mediate antidiabetic metabolic effects. Nature 423(6941): 762-769. doi:10.1038/nature01705. PubMed: 12802337. 12802337

[27] Evans RM, Barish GD, Wang YX (2004) PPARs and the complex journey to obesity. Nat Med 10(4): 355-361. doi:10.1038/nm1025. PubMed: 15057233. 15057233

[28] Awazawa M, Ueki K, Inabe K, Yamauchi T, Kubota N et al. (2011) Adiponectin enhances insulin sensitivity by increasing hepatic IRS-2 expression via a macrophage-derived IL-6-dependent pathway. Cell Metab 13(4): 401-412. doi:10.1016/j.cmet.2011.02.010. PubMed: 21459325. 21459325

[29] Schadinger SE, Bucher NL, Schreiber BM, Farmer SR (2005) PPARgamma2 regulates lipogenesis and lipid accumulation in steatotic hepatocytes. Am J Physiol Endocrinol Metab 288(6): E1195-E1205. doi:10.1152/ajpendo.00513.2004. PubMed: 15644454. 15644454

[30] White MF (1997) The insulin signalling system and the IRS proteins. Diabetologia 40 Suppl 2: S2-17. doi:10.1007/s001250051387. PubMed: 9248696. 9248696

[31] Hosoe T, Nakahama T, Inouye Y (2005) Divergent Modes of Induction of Rat Hepatic and Pulmonary CYP3A1 by Dexamethasone and Pregnenolone 16α-Carbonitrile. J Health Sci 51(1): 75-79. doi:10.1248/jhs.51.75.

[32] Saccò M, Valenti G, Corvi Mora P, Wu FC, Ray DW (2002) DHEA, a selective glucocorticoid receptor antagonist: its role in immune system regulation and metabolism. J Endocrinol Invest 25(10 Suppl): 81-82. PubMed: 12508928. 12508928

[33] Lähteenmäki P, Ylöstalo P, Sipinen S, Toivonen J, Ruusuvaara L et al. (1980) Return of ovulation after abortion and after discontinuation of oral contraceptives. Fertil Steril 34(3): 246-249. PubMed: 7409246. 7409246 10.1016/s0015-0282(16)44956-3

[34] Feldman D, Couropmitree C (1976) Intrinsic mineralocorticoid agonist activity of some nonsteroidal anti-inflammatory drugs. A postulated mechanism for sodium retention. J Clin Invest 57(1): 1-7. doi:10.1172/JCI108249. PubMed: 173739. 173739 PMC436618

[35] Lin S, Han Y, Shi Y, Rong H, Zheng S et al. (2012) Revealing a steroid receptor ligand as a unique PPARgamma agonist. Cell Res 22(4): 746-756. doi:10.1038/cr.2011.162. PubMed: 21986665. 21986665 PMC3257359

[36] Moriuchi A, Yamasaki H, Shimamura M, Kita A, Kuwahara H et al. (2007) Induction of human adiponectin gene transcription by telmisartan, angiotensin receptor blocker, independently on PPAR-gamma activation. Biochem Biophys Res Commun 356(4): 1024-1030. doi:10.1016/j.bbrc.2007.03.084. PubMed: 17399685. 17399685

[37] Lowe CE, O'Rahilly S, Rochford JJ (2011) Adipogenesis at a glance. J Cell Sci 124(16): 2681-2686. doi:10.1242/jcs.079699. 21807935

[38] Banga A, Unal R, Tripathi P, Pokrovskaya I, Owens RJ et al. (2009) Adiponectin translation is increased by the PPARgamma agonists pioglitazone and omega-3 fatty acids. Am J Physiol Endocrinol Metab 296(3): E480-E489. PubMed: 19088251. 19088251 10.1152/ajpendo.90892.2008 PMC2660148

[39] Ota T, Takamura T, Kurita S, Matsuzawa N, Kita Y et al. (2007) Insulin resistance accelerates a dietary rat model of nonalcoholic steatohepatitis. Gastroenterology 132(1): 282-293. doi:10.1053/j.gastro.2006.10.014. PubMed: 17241878. 17241878

[40] Wu X, Motoshima H, Mahadev K, Stalker TJ, Scalia R et al. (2003) Involvement of AMP-activated protein kinase in glucose uptake stimulated by the globular domain of adiponectin in primary rat adipocytes. Diabetes 52(6): 1355-1363. doi:10.2337/diabetes.52.6.1355. PubMed: 12765944. 12765944

[41] Okuno A, Tamemoto H, Tobe K, Ueki K, Mori Y et al. (1998) Troglitazone increases the number of small adipocytes without the change of white adipose tissue mass in obese Zucker rats. J Clin Invest 101(6): 1354-1361. doi:10.1172/JCI1235. PubMed: 9502777. 9502777 PMC508690

[42] Hasan AU, Ohmori K, Hashimoto T, Kamitori K, Hirata Y et al. (2011) Pioglitazone promotes preadipocyte proliferation by downregulating p16(Ink4a). Biochem Biophys Res Commun 411(2): 375-380. doi:10.1016/j.bbrc.2011.06.152. PubMed: 21741366. 21741366

[43] Saltiel AR, Olefsky JM (1996) Thiazolidinediones in the treatment of insulin resistance and type II diabetes. Diabetes 45(12): 1661-1669. doi:10.2337/diab.45.12.1661. PubMed: 8922349. 8922349

[44] Musgrove EA, Lee CS, Cornish AL, Swarbrick A, Sutherland RL (1997) Antiprogestin inhibition of cell cycle progression in T-47D breast cancer cells is accompanied by induction of the cyclin-dependent kinase inhibitor p21. Mol Endocrinol 11(1): 54-66. doi:10.1210/me.11.1.54. PubMed: 8994188. 8994188

[45] Blanquicett C, Roman J, Hart CM (2008) Thiazolidinediones as anti-cancer agents. Cancer Ther 6: 25–34(A):25-34 PubMed: 19079765. PMC2600565 19079765

[46] Wang Y, Lu G, Wong WP, Vliegenthart JF, Gerwig GJ et al. (2004) Proteomic and functional characterization of endogenous adiponectin purified from fetal bovine serum. Proteomics 4(12): 3933-3942. doi:10.1002/pmic.200400826. PubMed: 15378692. 15378692

