# Supplementary material for: Mifepristone Promotes Adiponectin Production and Improves Insulin Sensitivity in a Mouse Model of Diet-Induced-Obesity
Source: PLoS One. 2013 Nov 6;8(11):e79724. doi: 10.1371/journal.pone.0079724 (PMC3819252; doi:10.1371/journal.pone.0079724)
Supplement: Figure S1 — Effects of mifepristone on perirenal adipose tissues in HFD mice. Mice had been fed with HFD and mifepristone as described above. Perirenal adipose tissues was collected when the animals were sacrificed. Panel shows the results of qRT-PCR assays in perirenal adipose tissue of HFD-induced obesity mice treated with or without mifepristone. Total RNA was isolated and subjected to qRT-PCR using primers and probes directed to aP2, FAS, leptin, and PPARγ. Expression level of each transcript was normalized to that of 18S. Shown are the results derived from pooled data, plotting the fold increase of the expression levels of indicated gene at the mifepristone-concentration indicated, relative to the signals obtained in the absence of mifepristone (HFD alone). Each data represents the mean ± S.E.M. derived from 8 independent experiments. * p < 0.05, ** p < 0.01 versus HFD fed mice that did not receive mifepristone. (PPT) [file pone.0079724.s001.ppt]

## Slide 1
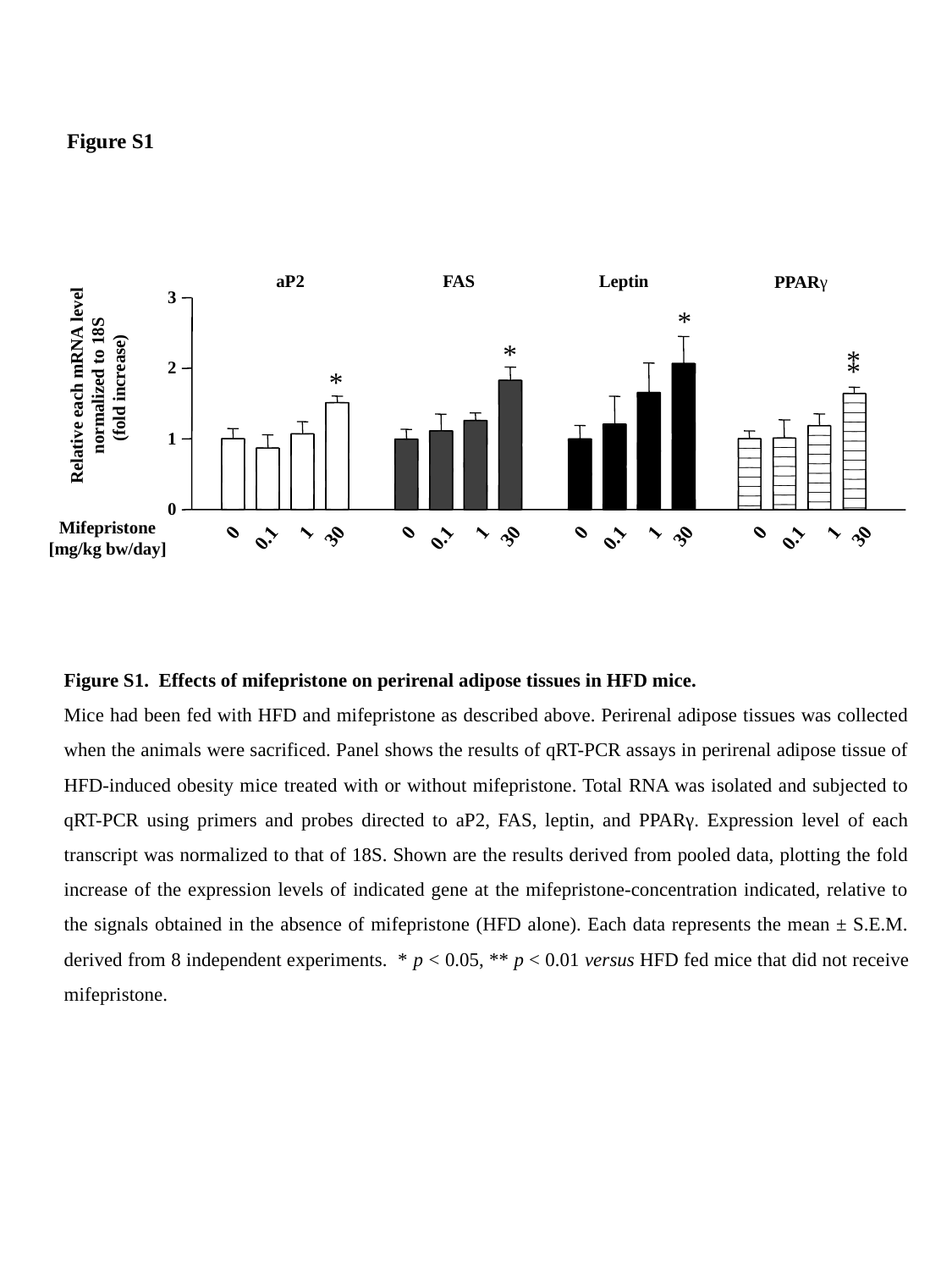

Figure S1
aP2
FAS
Leptin
PPARγ
*
*
*
*
*
0
1
0
1
0
1
0
1
30
30
30
30
0.1
0.1
0.1
0.1
3
Relative each mRNA level normalized to 18S
(fold increase)
2
1
0
Mifepristone
[mg/kg bw/day]
Figure S1. Effects of mifepristone on perirenal adipose tissues in HFD mice.
Mice had been fed with HFD and mifepristone as described above. Perirenal adipose tissues was collected when the animals were sacrificed. Panel shows the results of qRT-PCR assays in perirenal adipose tissue of HFD-induced obesity mice treated with or without mifepristone. Total RNA was isolated and subjected to qRT-PCR using primers and probes directed to aP2, FAS, leptin, and PPARγ. Expression level of each transcript was normalized to that of 18S. Shown are the results derived from pooled data, plotting the fold increase of the expression levels of indicated gene at the mifepristone-concentration indicated, relative to the signals obtained in the absence of mifepristone (HFD alone). Each data represents the mean ± S.E.M. derived from 8 independent experiments. * p < 0.05, ** p < 0.01 versus HFD fed mice that did not receive mifepristone.
