# Supplementary material for: Mifepristone Promotes Adiponectin Production and Improves Insulin Sensitivity in a Mouse Model of Diet-Induced-Obesity
Source: PLoS One. 2013 Nov 6;8(11):e79724. doi: 10.1371/journal.pone.0079724 (PMC3819252; doi:10.1371/journal.pone.0079724)
Supplement: Figure S2 — Effects of mifepristone on percent of total body weight (Liver and Perirenal adipose tissue) in HFD mice. Tissue weights (liver and perirenal adipose tissues) in HFD induced obese mice are expressed as percentage of body weight. Each data represents the mean ± S.E.M. derived from 8 independent experiments. * p < 0.05 versus HFD fed mice that did not receive mifepristone. (PPT) [file pone.0079724.s002.ppt]

## Slide 1
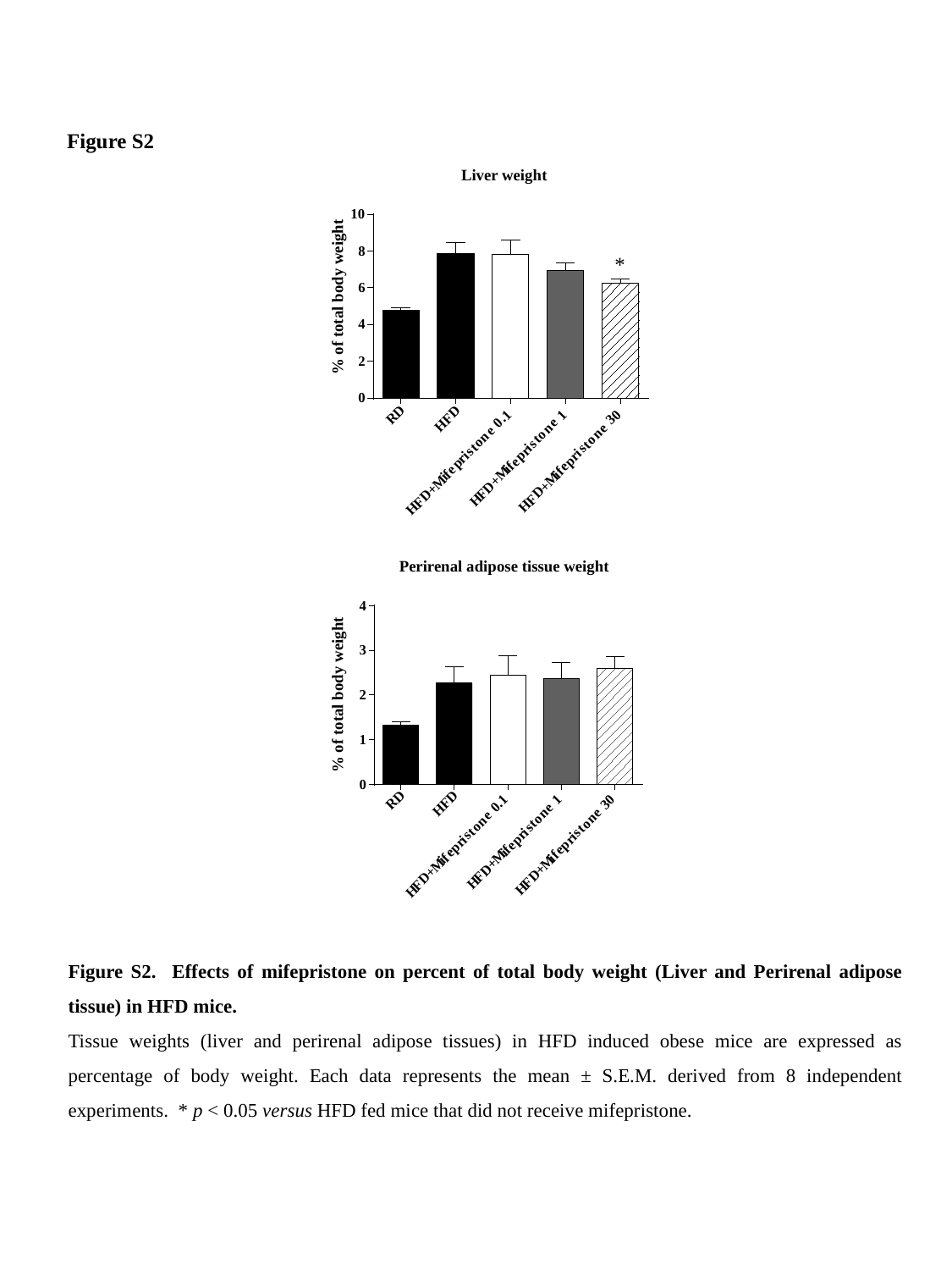

Figure S2
Liver weight
10
8
6
4
2
0
D
D
1
1
0
.
F
R
3
0
e
H
n
e
e
n
o
n
t
o
o
s
t
t
i
s
r
s
i
i
r
p
r
p
e
p
f
e
i
e
f
M
f
i
i
M
+
M
+
D
+
D
F
D
H
F
F
H
H
*
% of total body weight
Perirenal adipose tissue weight
4
3
2
1
0
D
D
1
1
0
.
F
R
3
0
e
H
n
e
e
n
o
n
t
o
o
s
t
t
i
s
r
s
i
i
r
p
r
p
e
p
f
e
i
e
f
M
f
i
i
M
+
M
+
D
+
D
F
D
F
H
F
H
H
% of total body weight
Figure S2. Effects of mifepristone on percent of total body weight (Liver and Perirenal adipose tissue) in HFD mice.
Tissue weights (liver and perirenal adipose tissues) in HFD induced obese mice are expressed as percentage of body weight. Each data represents the mean ± S.E.M. derived from 8 independent experiments. * p < 0.05 versus HFD fed mice that did not receive mifepristone.
