# Supplementary material for: Mifepristone Promotes Adiponectin Production and Improves Insulin Sensitivity in a Mouse Model of Diet-Induced-Obesity
Source: PLoS One. 2013 Nov 6;8(11):e79724. doi: 10.1371/journal.pone.0079724 (PMC3819252; doi:10.1371/journal.pone.0079724)
Supplement: Figure S3 — Effects of mifepristone on matured adipocytes. Shown are the results of qRT-PCR and immunoblot analysis. Cells were treated with 0.1 and 1 µM of mifepristone for the times indicated. Upper panel shows that the expression level of adiponectin mRNA was normalized to that of 18S, respectively. Shown are the results derived from pooled data, plotting the fold increase of the degree of expression level of adiponectin mRNA, relative to the values obtained in the absence of mifepristone (day 0). Each data represents the mean ± S.E.M. derived from 4 independent experiments. * p < 0.05, ** p < 0.01 versus the absence of mifepristone. Lower panel shows adiponectin secretion levels from cells kept for 3 days after indicated concentration of mifepristone. (PPT) [file pone.0079724.s003.ppt]

## Slide 1
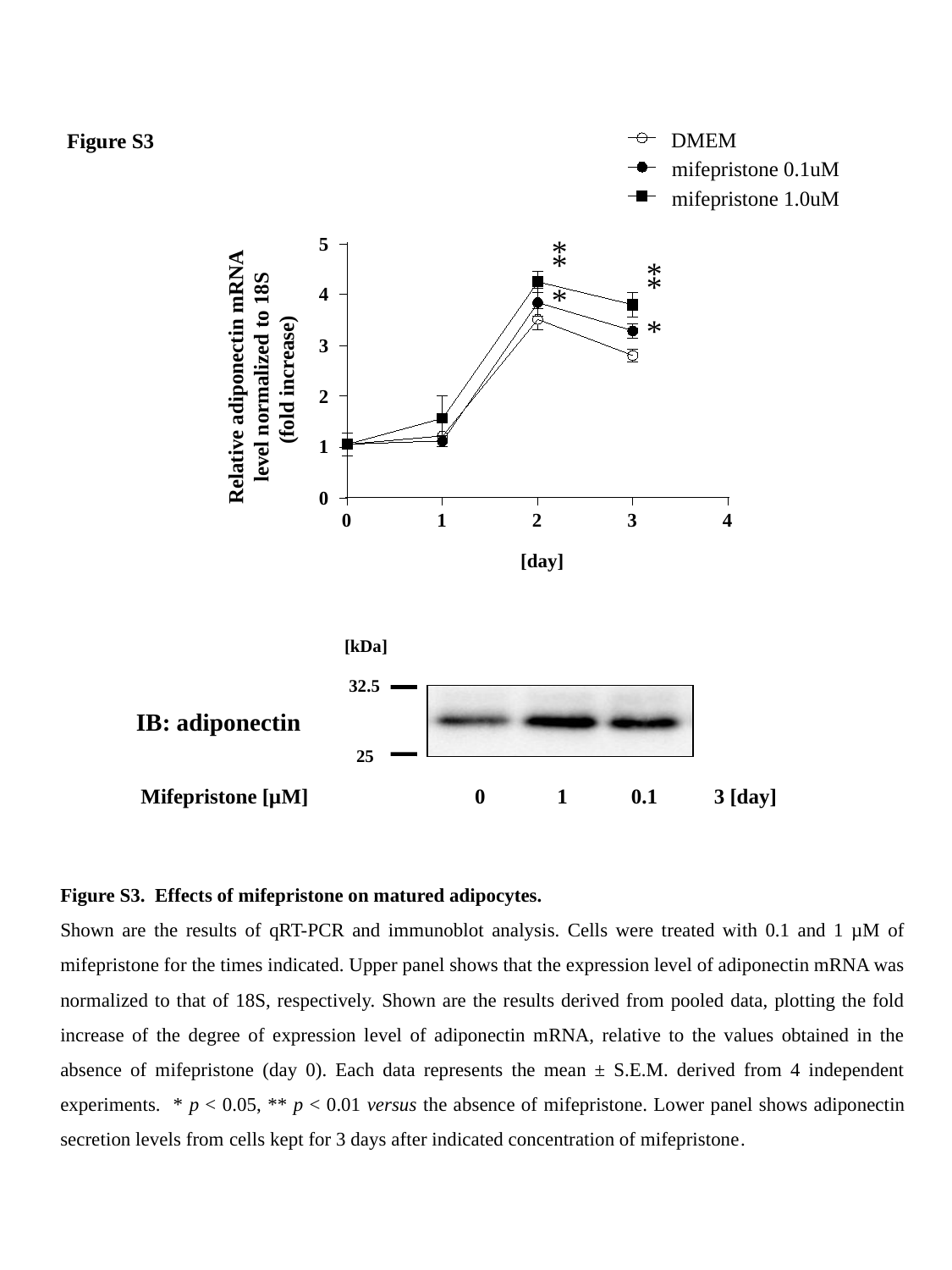

Figure S3
DMEM
mifepristone 0.1uM
mifepristone 1.0uM
*
5
*
*
*
*
4
*
3
Relative adiponectin mRNA level normalized to 18S
(fold increase)
2
1
0
0
1
2
3
4
[day]
[kDa]
32.5
IB: adiponectin
25
Mifepristone [µM]
0
1
0.1
3 [day]
Figure S3. Effects of mifepristone on matured adipocytes.
Shown are the results of qRT-PCR and immunoblot analysis. Cells were treated with 0.1 and 1 µM of mifepristone for the times indicated. Upper panel shows that the expression level of adiponectin mRNA was normalized to that of 18S, respectively. Shown are the results derived from pooled data, plotting the fold increase of the degree of expression level of adiponectin mRNA, relative to the values obtained in the absence of mifepristone (day 0). Each data represents the mean ± S.E.M. derived from 4 independent experiments. * p < 0.05, ** p < 0.01 versus the absence of mifepristone. Lower panel shows adiponectin secretion levels from cells kept for 3 days after indicated concentration of mifepristone.
