# Supplementary material for: Mifepristone Promotes Adiponectin Production and Improves Insulin Sensitivity in a Mouse Model of Diet-Induced-Obesity
Source: PLoS One. 2013 Nov 6;8(11):e79724. doi: 10.1371/journal.pone.0079724 (PMC3819252; doi:10.1371/journal.pone.0079724)
Supplement: Figure S4 — Effects of mifepristone and PPARγ antagonists on total cellular protein level in matured adipocytes. Shown are the results of total cellular protein. Cells were treated with 0.1 and 1 µM of mifepristone. Total cellular proteins were extracted using a buffer containing n-octyl b-glucopyranoside. Protein determinations were made with Bicinchoninate Protein Assay Kit (Nacalai, Kyoto, Japan) with bovine serum albumin as a standard. Shown are the results derived from pooled data, relative to the values obtained in the absence of mifepristone (day 3). Each data represents the mean ± S.E.M. derived from 4 independent experiments. (PPT) [file pone.0079724.s004.ppt]

## Slide 1
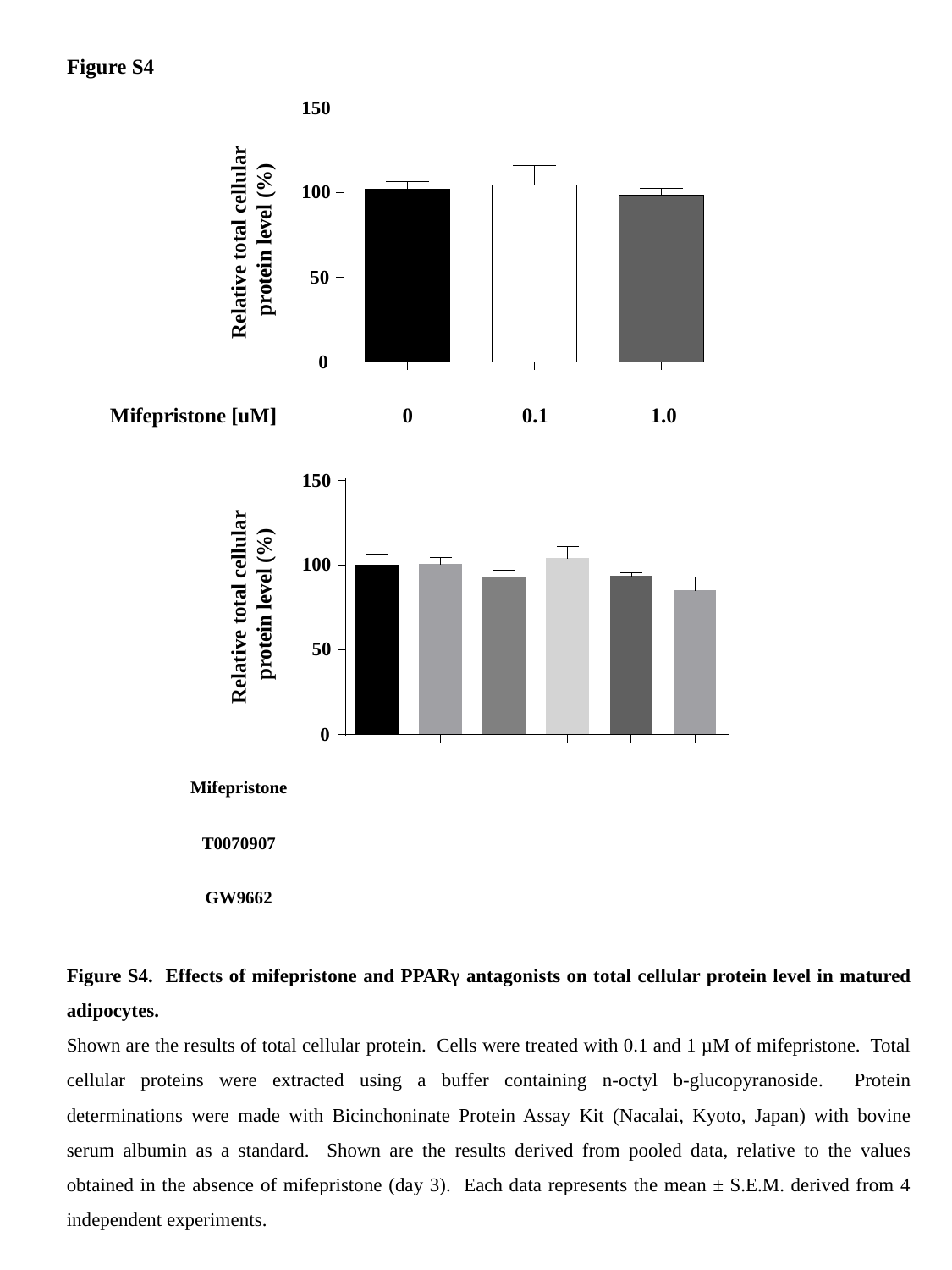

Figure S4
150
100
50
0
Relative total cellular
protein level (%)
Mifepristone [uM]
0
0.1
1.0
150
100
50
0
Relative total cellular
protein level (%)
－
＋
＋
－
＋
－
Mifepristone
－
－
＋
＋
－
－
T0070907
－
－
－
－
＋
＋
GW9662
Figure S4. Effects of mifepristone and PPARγ antagonists on total cellular protein level in matured adipocytes.
Shown are the results of total cellular protein. Cells were treated with 0.1 and 1 µM of mifepristone. Total cellular proteins were extracted using a buffer containing n-octyl b-glucopyranoside. Protein determinations were made with Bicinchoninate Protein Assay Kit (Nacalai, Kyoto, Japan) with bovine serum albumin as a standard. Shown are the results derived from pooled data, relative to the values obtained in the absence of mifepristone (day 3). Each data represents the mean ± S.E.M. derived from 4 independent experiments.
