# Supplementary material for: Mifepristone Promotes Adiponectin Production and Improves Insulin Sensitivity in a Mouse Model of Diet-Induced-Obesity
Source: PLoS One. 2013 Nov 6;8(11):e79724. doi: 10.1371/journal.pone.0079724 (PMC3819252; doi:10.1371/journal.pone.0079724)
Supplement: Figure S5 — Effects of mifepristone on cell number in matured adipocytes. The degrees of cell number were determined using 4′,6-diamidino-2-phenylindole (DAPI) solution, visualized nuclear DNA. Cells were fixed, stained and observed by microscope (BioRevo, KEYENCE). Shown are the results derived from pooled data, relative to the values obtained in the absence of mifepristone (day 3). Each data represents the mean ± S.E.M. derived from 3 independent experiments. Bar, 100 microm. (PPT) [file pone.0079724.s005.ppt]

## Slide 1
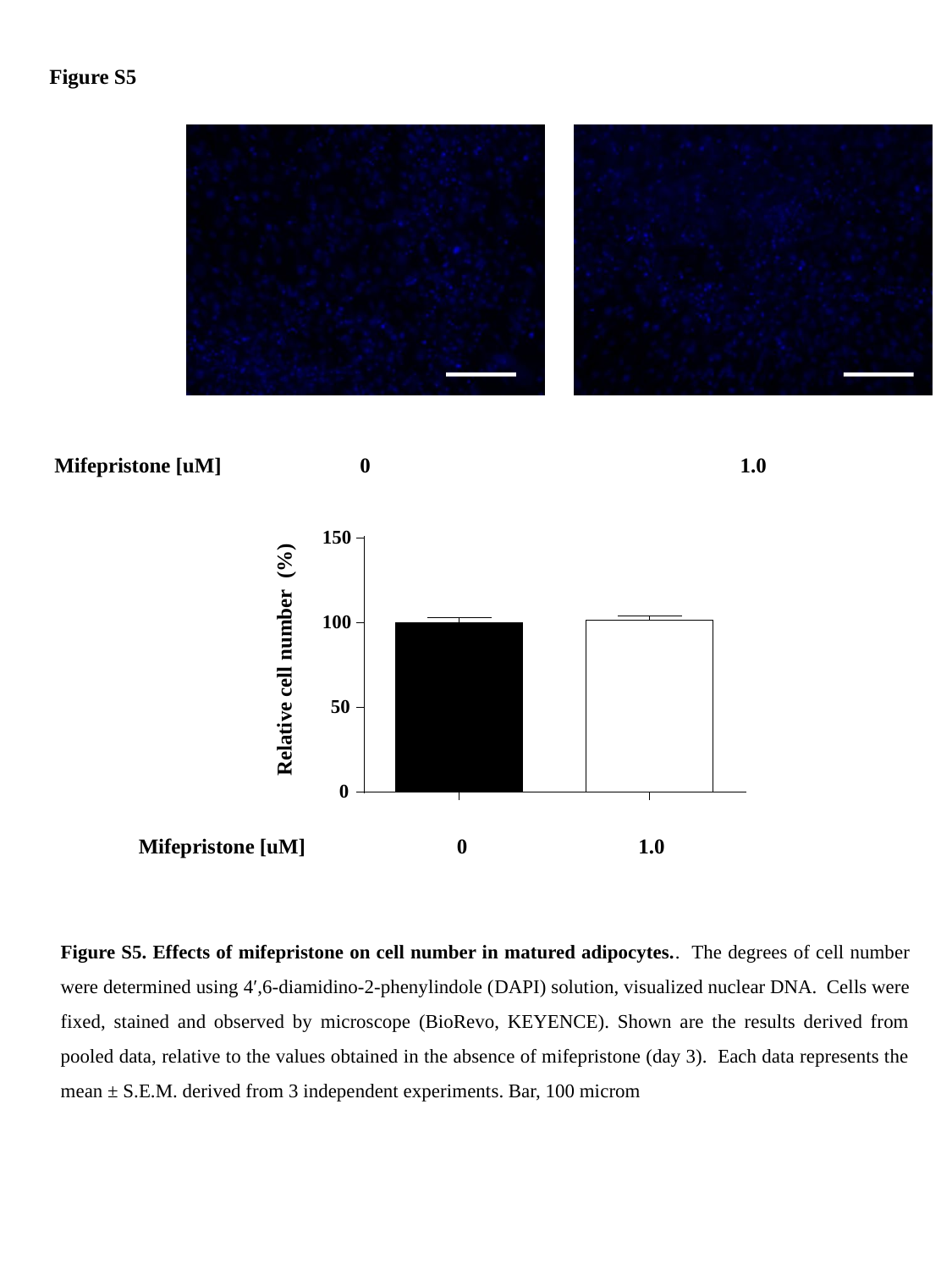

Figure S5
Mifepristone [uM]
0
1.0
150
100
50
0
Relative cell number (%)
Mifepristone [uM]
0
1.0
Figure S5. Effects of mifepristone on cell number in matured adipocytes.. The degrees of cell number were determined using 4′,6-diamidino-2-phenylindole (DAPI) solution, visualized nuclear DNA. Cells were fixed, stained and observed by microscope (BioRevo, KEYENCE). Shown are the results derived from pooled data, relative to the values obtained in the absence of mifepristone (day 3). Each data represents the mean ± S.E.M. derived from 3 independent experiments. Bar, 100 microm
