# Supplementary material for: Mifepristone Promotes Adiponectin Production and Improves Insulin Sensitivity in a Mouse Model of Diet-Induced-Obesity
Source: PLoS One. 2013 Nov 6;8(11):e79724. doi: 10.1371/journal.pone.0079724 (PMC3819252; doi:10.1371/journal.pone.0079724)
Supplement: Figure S6 — Immunoblot analysis of adiponectin secretion levels into culture medium (DMEM) with or without fetal bovine serum (FBS). Cells were kept for 3 days after mifepristone stimulation, then equal volume of medium containing 2x Laemmli sample buffer was added. * p < 0.05 versus vehicle, Each value and vertical bar represents the mean±SE (n = 10). (PPT) [file pone.0079724.s006.ppt]

## Slide 1
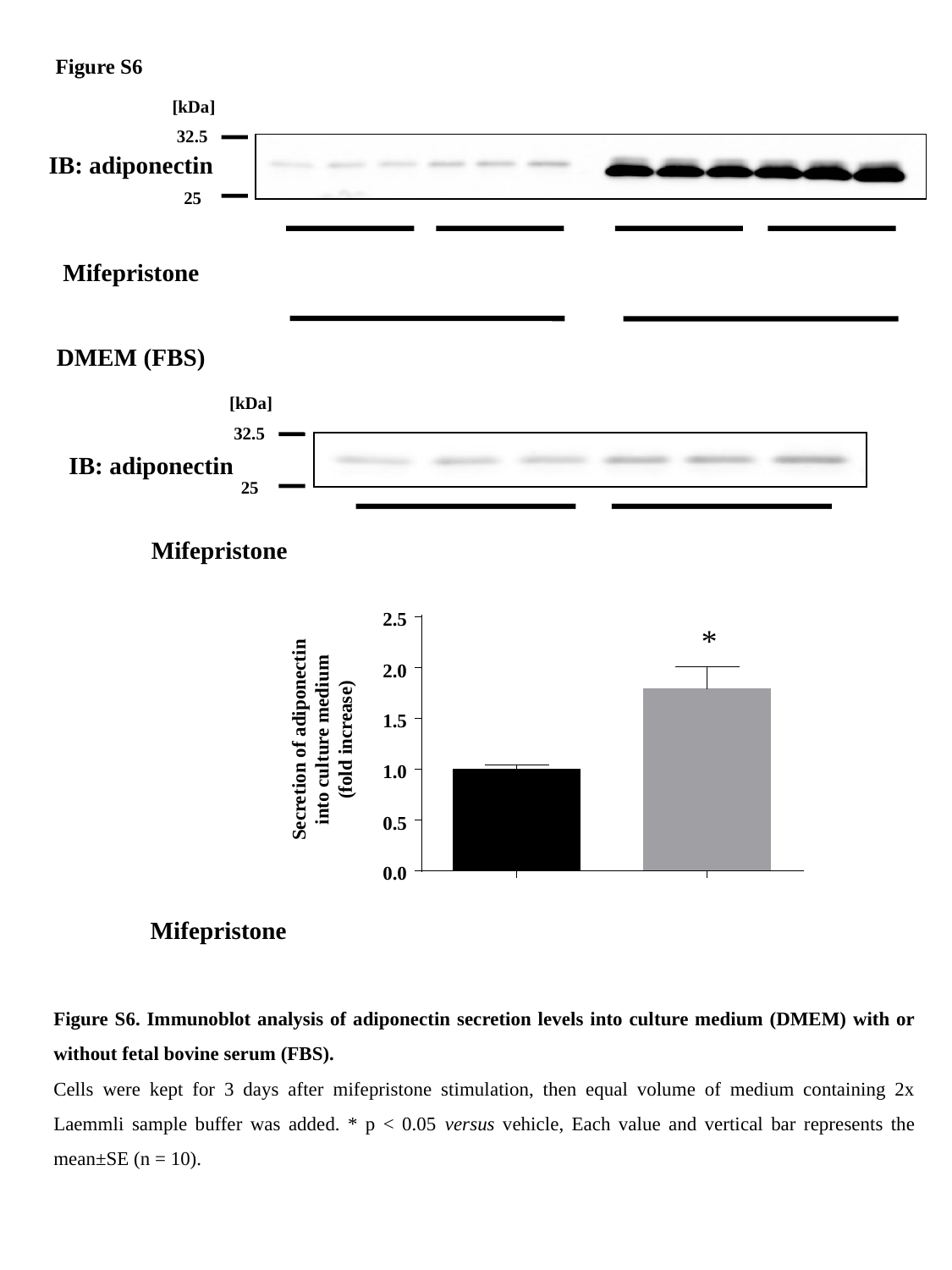

Figure S6
[kDa]
32.5
IB: adiponectin
25
Mifepristone
－
＋
－
＋
DMEM (FBS)
－
＋
[kDa]
32.5
IB: adiponectin
25
Mifepristone
－
＋
2.5
*
2.0
Secretion of adiponectin
into culture medium
(fold increase)
1.5
1.0
0.5
0.0
Mifepristone
－
＋
Figure S6. Immunoblot analysis of adiponectin secretion levels into culture medium (DMEM) with or without fetal bovine serum (FBS).
Cells were kept for 3 days after mifepristone stimulation, then equal volume of medium containing 2x Laemmli sample buffer was added. * p < 0.05 versus vehicle, Each value and vertical bar represents the mean±SE (n = 10).
