# Supplementary material for: Mifepristone Promotes Adiponectin Production and Improves Insulin Sensitivity in a Mouse Model of Diet-Induced-Obesity
Source: PLoS One. 2013 Nov 6;8(11):e79724. doi: 10.1371/journal.pone.0079724 (PMC3819252; doi:10.1371/journal.pone.0079724)
Supplement: Figure S7 — Differentiated 3T3-L1 adipocytes had been transiently transfected with control or siRNA targeted to PPARγ 48 hours prior to mifepristone stimulation. Cells were fixed and observed by microscope (BioRevo, KEYENCE). Bar, 100 microm. (PPT) [file pone.0079724.s007.ppt]

## Slide 1
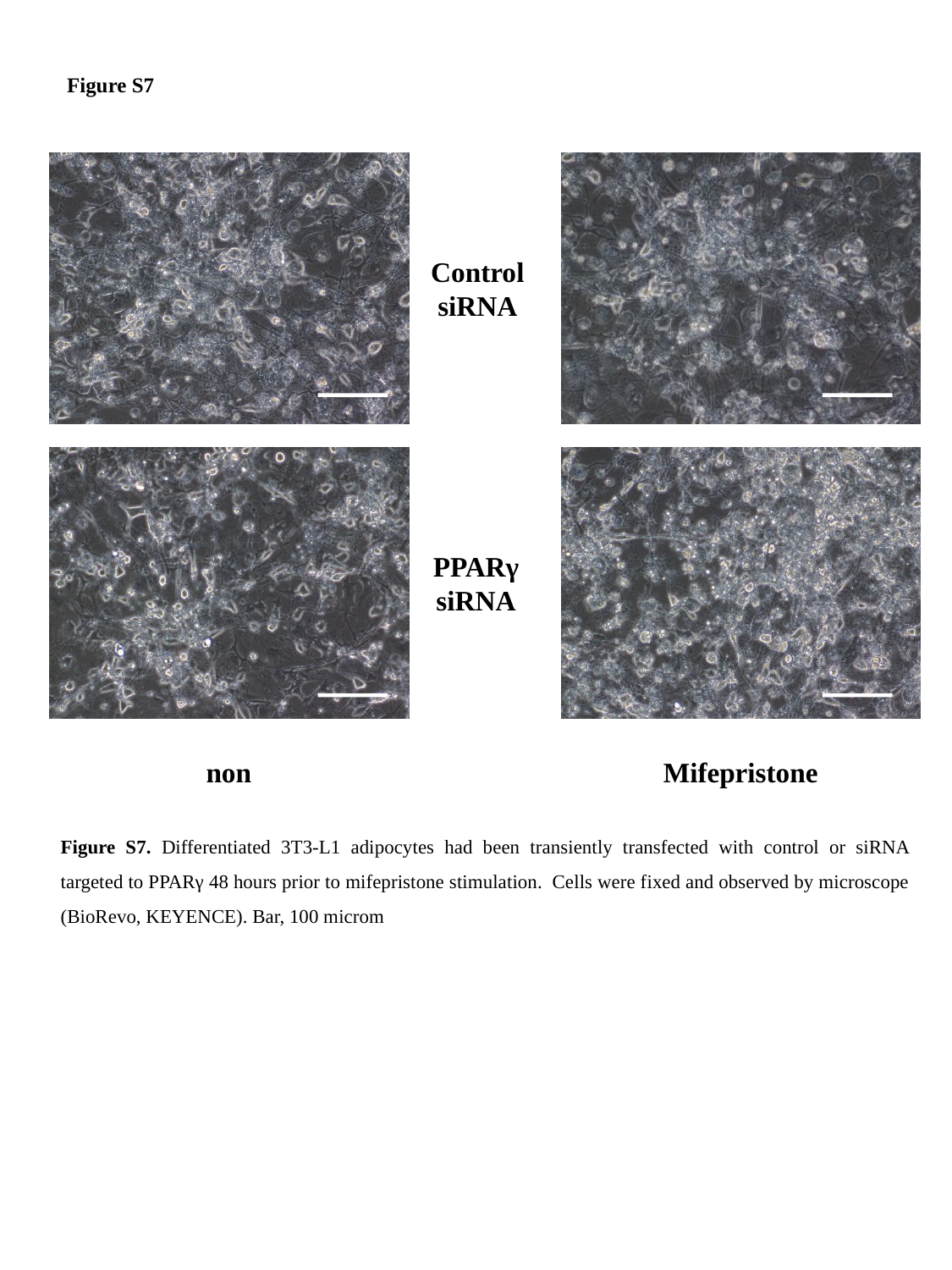

Figure S7
Control
siRNA
PPARγ
siRNA
non
Mifepristone
Figure S7. Differentiated 3T3-L1 adipocytes had been transiently transfected with control or siRNA targeted to PPARγ 48 hours prior to mifepristone stimulation. Cells were fixed and observed by microscope (BioRevo, KEYENCE). Bar, 100 microm
