# Supplementary material for: Mifepristone Promotes Adiponectin Production and Improves Insulin Sensitivity in a Mouse Model of Diet-Induced-Obesity
Source: PLoS One. 2013 Nov 6;8(11):e79724. doi: 10.1371/journal.pone.0079724 (PMC3819252; doi:10.1371/journal.pone.0079724)
Supplement: Figure S8 — Effects of mifepristone on total cellular protein level in matured adipocytes transfected with each siRNA. Shown are the results of total cellular protein. Cells were transfected with siRNA for control or PPARγ treated with/without mifepristone. Total cellular proteins were extracted using a buffer containing n-octyl b-glucopyranoside. Protein determinations were made with Bicinchoninate Protein Assay Kit (Nacalai, Kyoto, Japan) with bovine serum albumin as a standard. Shown are the results derived from pooled data, relative to the values obtained in the absence of mifepristone (day 3). Each data represents the mean ± S.E.M. derived from 4 independent experiments. (PPT) [file pone.0079724.s008.ppt]

## Slide 1
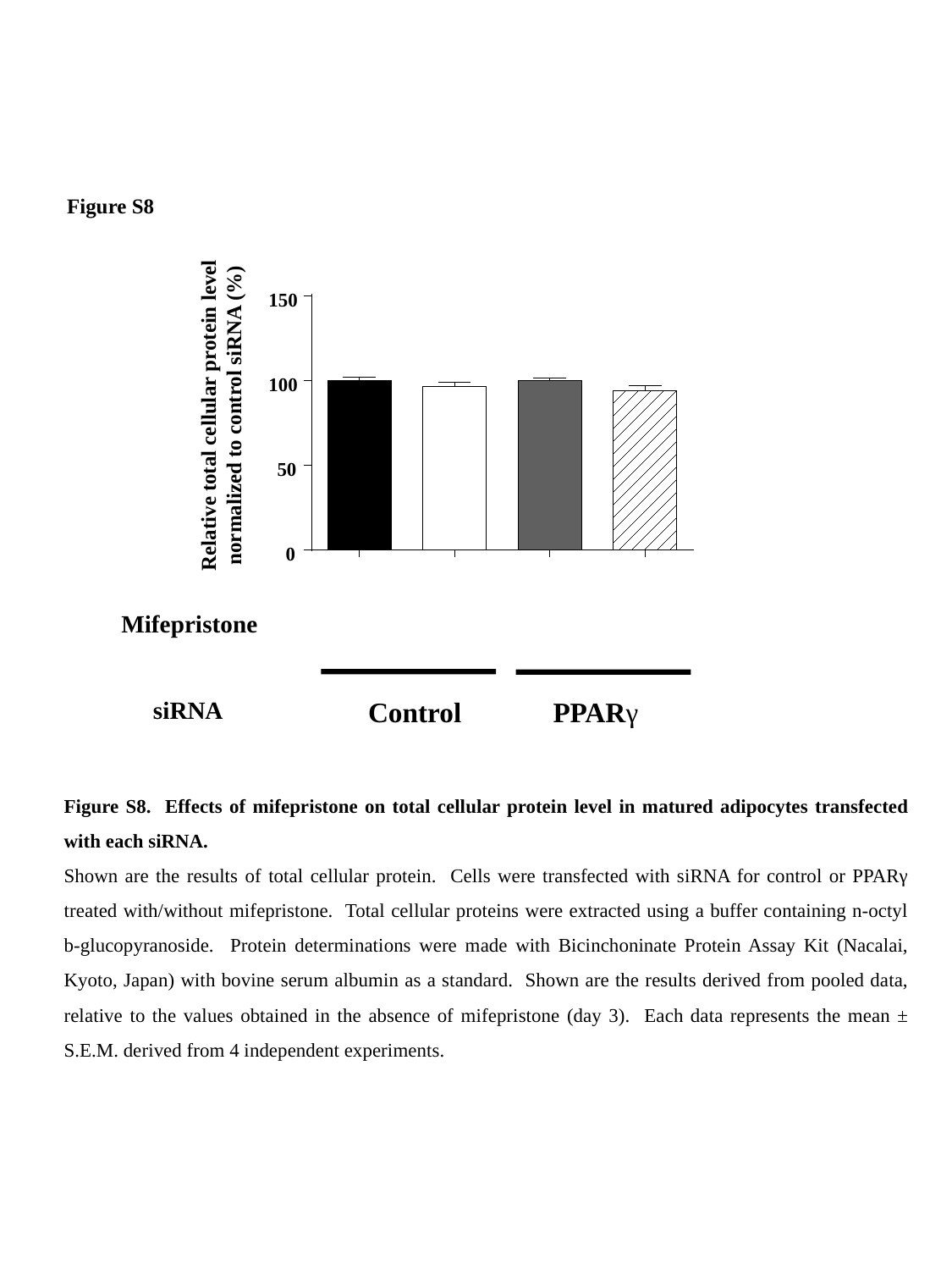

Figure S8
150
100
50
0
Relative total cellular protein level normalized to control siRNA (%)
－
＋
－
＋
Mifepristone
siRNA
Control
PPARγ
Figure S8. Effects of mifepristone on total cellular protein level in matured adipocytes transfected with each siRNA.
Shown are the results of total cellular protein. Cells were transfected with siRNA for control or PPARγ treated with/without mifepristone. Total cellular proteins were extracted using a buffer containing n-octyl b-glucopyranoside. Protein determinations were made with Bicinchoninate Protein Assay Kit (Nacalai, Kyoto, Japan) with bovine serum albumin as a standard. Shown are the results derived from pooled data, relative to the values obtained in the absence of mifepristone (day 3). Each data represents the mean ± S.E.M. derived from 4 independent experiments.
