# Supplementary material for: Mifepristone Promotes Adiponectin Production and Improves Insulin Sensitivity in a Mouse Model of Diet-Induced-Obesity
Source: PLoS One. 2013 Nov 6;8(11):e79724. doi: 10.1371/journal.pone.0079724 (PMC3819252; doi:10.1371/journal.pone.0079724)
Supplement: Table S1 — Parameters of regular diet mice. Seven-week-old C57BL/6NCr Slc mice received regular diet (RD) and were orally treated with either mifepristone (0.1, 1 or 30 mg/kg bw/day) or vehicle (RD alone) for twenty-two consecutive weeks (n = 8 in each group). At the age of week 28, animals were subjected to fasting blood glucose test. * p < 0.05, ** p < 0.01 versus RD fed mice that did not receive mifepristone. (PPT) [file pone.0079724.s009.ppt]

## Slide 1
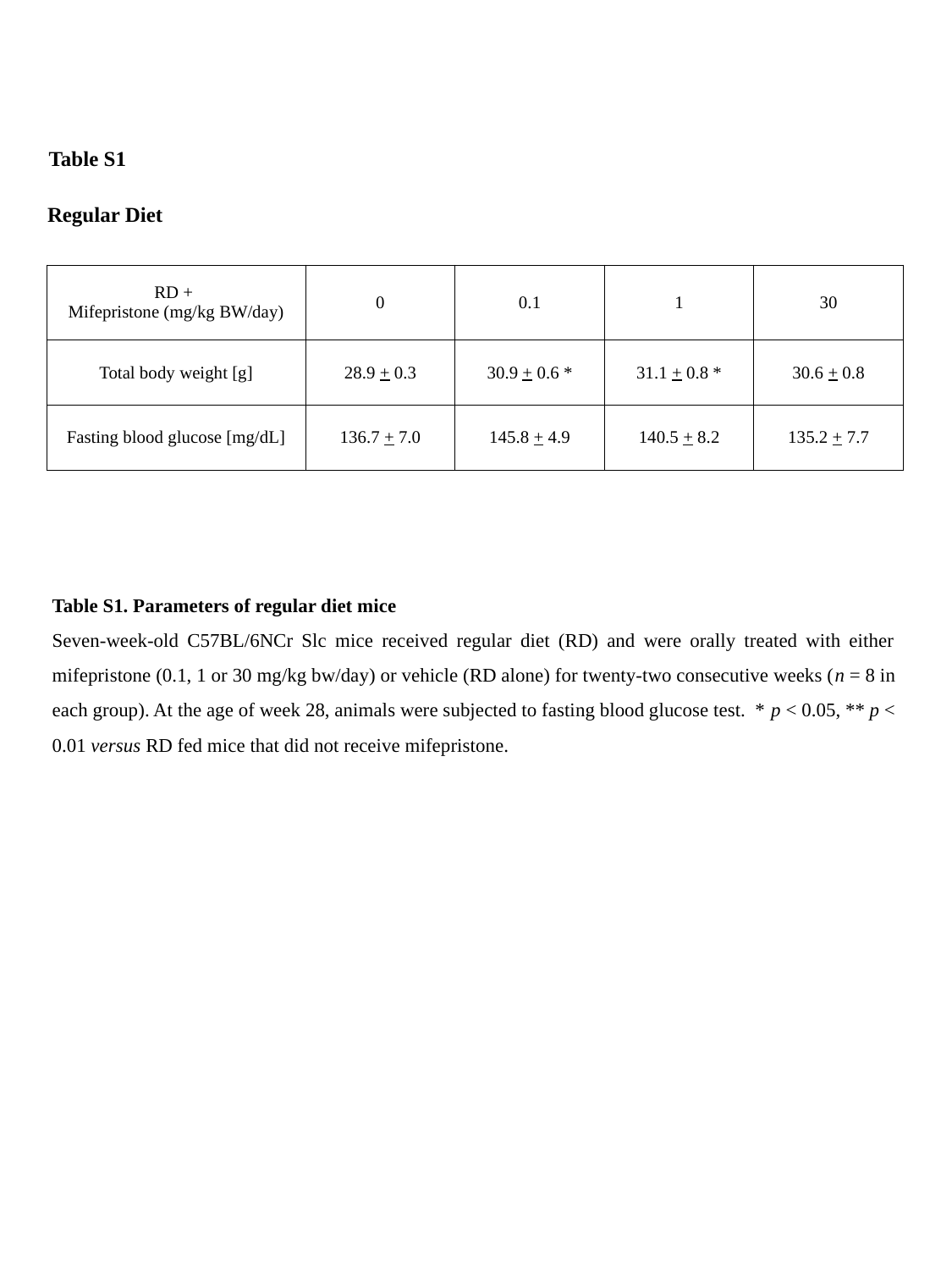

Table S1
Regular Diet
| RD + Mifepristone (mg/kg BW/day) | 0 | 0.1 | 1 | 30 |
| --- | --- | --- | --- | --- |
| Total body weight [g] | 28.9 + 0.3 | 30.9 + 0.6 \* | 31.1 + 0.8 \* | 30.6 + 0.8 |
| Fasting blood glucose [mg/dL] | 136.7 + 7.0 | 145.8 + 4.9 | 140.5 + 8.2 | 135.2 + 7.7 |
Table S1. Parameters of regular diet mice
Seven-week-old C57BL/6NCr Slc mice received regular diet (RD) and were orally treated with either mifepristone (0.1, 1 or 30 mg/kg bw/day) or vehicle (RD alone) for twenty-two consecutive weeks (n = 8 in each group). At the age of week 28, animals were subjected to fasting blood glucose test. * p < 0.05, ** p < 0.01 versus RD fed mice that did not receive mifepristone.
