# Supplementary material for: Mifepristone Promotes Adiponectin Production and Improves Insulin Sensitivity in a Mouse Model of Diet-Induced-Obesity
Source: PLoS One. 2013 Nov 6;8(11):e79724. doi: 10.1371/journal.pone.0079724 (PMC3819252; doi:10.1371/journal.pone.0079724)
Supplement: Table S2 — LDH activity assay. LDH activity of culture medium was measured using a CytoTox-ONE Homogenous Membrane Integrity Assay kit (Promega), using DMEM and cell lysates extracted with a lysis buffer containing Triton-X (0.1% v/v) as negative and positive controls, respectively. (PPT) [file pone.0079724.s010.ppt]

## Slide 1
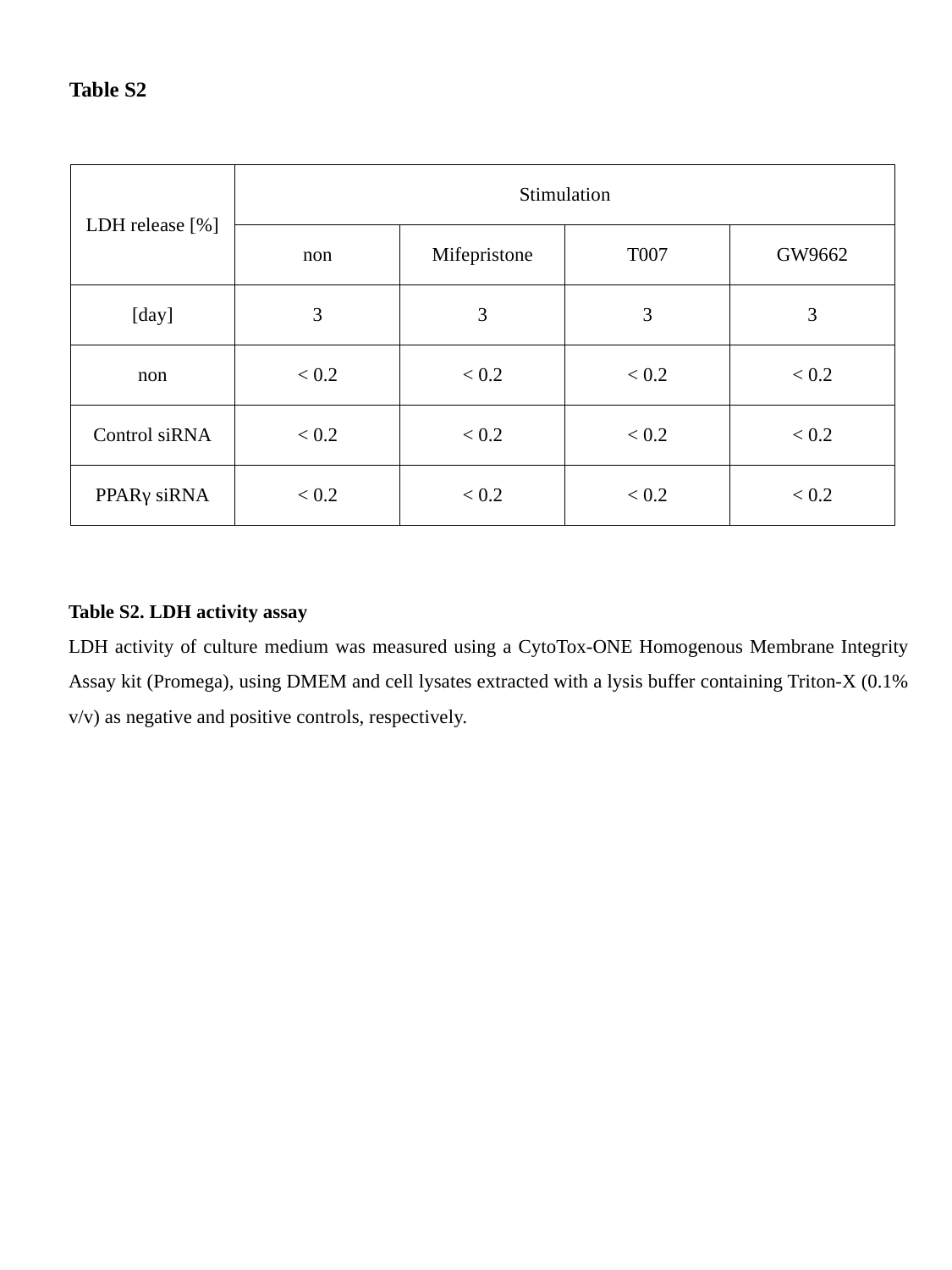

Table S2
| LDH release [%] | Stimulation | | | |
| --- | --- | --- | --- | --- |
| | non | Mifepristone | T007 | GW9662 |
| [day] | 3 | 3 | 3 | 3 |
| non | < 0.2 | < 0.2 | < 0.2 | < 0.2 |
| Control siRNA | < 0.2 | < 0.2 | < 0.2 | < 0.2 |
| PPARγ siRNA | < 0.2 | < 0.2 | < 0.2 | < 0.2 |
Table S2. LDH activity assay
LDH activity of culture medium was measured using a CytoTox-ONE Homogenous Membrane Integrity Assay kit (Promega), using DMEM and cell lysates extracted with a lysis buffer containing Triton-X (0.1% v/v) as negative and positive controls, respectively.
